# Fabrication of r-GO/GO/α-Fe_2_O_3_/Fe_2_TiO_5_ Nanocomposite Using Natural Ilmenite and Graphite for Efficient Photocatalysis in Visible Light

**DOI:** 10.3390/ma16010139

**Published:** 2022-12-23

**Authors:** Leshan Usgodaarachchi, Madara Jayanetti, Charitha Thambiliyagodage, Heshan Liyanaarachchi, Saravanamuthu Vigneswaran

**Affiliations:** 1 Department of Materials Engineering, Faculty of Engineering, Sri Lanka Institute of Information Technology, Malabe 10115, Sri Lanka; 2 Faculty of Humanities and Sciences, Sri Lanka Institute of Information Technology, Malabe 10115, Sri Lanka; 3 Faculty of Engineering and Information Technology, University of Technology Sydney, P.O. Box 123, Broadway, Ultimo, NSW 2007, Australia; 4 Faculty of Sciences & Technology (RealTek), Norwegian University of Life Sciences, P.O. Box 5003, NO-1432 Ås, Norway

**Keywords:** ilmenite sand, visible light photocatalysis, reduced graphene oxide, type ii heterostructures, nanocomposites

## Abstract

Hematite (α-Fe_2_O_3_) and pseudobrookite (Fe_2_TiO_5_) suffer from poor charge transport and a high recombination effect under visible light irradiation. This study investigates the design and production of a 2D graphene-like r-GO/GO coupled α-Fe_2_O_3_/Fe_2_TiO_5_ heterojunction composite with better charge separation. It uses a simple sonochemical and hydrothermal approach followed by L-ascorbic acid chemical reduction pathway. The advantageous band offset of the α-Fe_2_O_3_/Fe_2_TiO_5_ (TF) nanocomposite between α-Fe_2_O_3_ and Fe_2_TiO_5_ forms a Type-II heterojunction at the Fe_2_O_3_/Fe_2_TiO_5_ interface, which efficiently promotes electron-hole separation. Importantly, very corrosive acid leachate resulting from the hydrochloric acid leaching of ilmenite sand, was successfully exploited to fabricate α-Fe_2_O_3_/Fe_2_TiO_5_ heterojunction. In this paper, a straightforward synthesis strategy was employed to create 2D graphene-like reduced graphene oxide (r-GO) from Ceylon graphite. The two-step process comprises oxidation of graphite to graphene oxide (GO) using the improved Hummer’s method, followed by controlled reduction of GO to r-GO using L-ascorbic acid. Before the reduction of GO to the r-GO, the surface of TF heterojunction was coupled with GO and was allowed for the controlled L-ascorbic acid reduction to yield r-GO/GO/α-Fe_2_O_3_/Fe_2_TiO_5_ nanocomposite. Under visible light illumination, the photocatalytic performance of the 30% GO/TF loaded composite material greatly improved (1240 Wcm^−2^). Field emission scanning electron microscopy (FE-SEM) and high-resolution transmission electron microscopy (HR-TEM) examined the morphological characteristics of fabricated composites. X-ray photoelectron spectroscopy (XPS), Raman, X-ray diffraction (XRD), X-ray fluorescence (XRF), and diffuse reflectance spectroscopy (DRS) served to analyze the structural features of the produced composites.

## 1. Introduction

Semiconductor photocatalysts have garnered a lot of attention for their versatility in a range of applications, such as environmental remediation [1], electronics [2], hydrogen production [3], medical uses [4], water purification [5], air purification [6], surface sterilization [7] and photochemical conversion [8]. Fujishima and Honda (1972) were the first to discover electrochemical water splitting by using TiO_2_ [9]. Accordingly, ZnO and SnO_2_ are considered alternatives to TiO_2_, used in various photocatalytic applications [10]. TiO_2_ exhibits two band gaps (Eg) of 3.2 and 3.0 eV for the anatase and rutile polymorphs, respectively [11]. According to the band energy values, calculated adsorption edges wavelengths for anatase and rutile are 387.45 and 413.28 nm, respectively, and represent the UV region excitation wavelengths. In the photocatalytic process, incident photons with energy greater than or equal to its bandgap energy (Eg) excitation of the semiconductor photocatalyst took place. That photoexcitation process caused electrons (e^−^) to be excited to the conduction band (CB), in order to participate in reduction reactions. Meanwhile holes (h^+^) remained in the valence band (VB) which enabled them to take part in oxidation reactions. Furthermore, the wide band gap of TiO_2_ led to light adsorption in the UV range by limiting the overall quantum efficiency of photoexcitation. However, for these broad band-gap semiconductors, only UV irradiation may be used, which accounts for just 4% of solar energy [12]. As a result, designing a material system able to use a broad range of the solar spectrum is critical for practical purposes. 

Many band-gap re-engineering strategies have been conducted to improve the photocatalytic efficiency of materials under visible light irradiation, such as metal doping [13], noble metal loading [14], non-metal loading [15], semiconductor coupling [16], heterojunction construction [17], and depositing high conductive graphene-like materials [18]. Of these, heterostructure formation with narrow bandgap transition metal oxides such as WO_3_ [19], VO_2_ [20], SnS [21], Fe_2_TiO_5_ [22], Fe_2_O_3_ [23], Fe_3_O_4_ [24], CuO [25], and Cu_2_O [26] emerged as an effective re-engineering prospect by facilitating photoexcitation in visible light irradiation. The synthesis of nanostructured photocatalytic materials has attracted much interest due to the significant advances in physicochemical properties [27,28,29]. Nanostructure fabrication increases the surface-to-volume ratio, which not only allows more photons to be harvested, resulting in more photoinduced electron-hole pairs but also provides shorter charge transport paths and more active sites on catalyst surfaces, resulting in faster reaction kinetics [30,31]. Furthermore, the construction of Type II and Type III band structures heterostructure plays an important role in effectively separating electrons and holes through the heterostructure [32,33]. However, these modifications induce the high quantum yield of photoexcited charge carriers. Nonetheless, the recombination of charge carriers can still be counted on the surface of photocatalysts with continuous destruction of quantum yield. Furthermore, the incorporation of charge carrier moveable materials such as reduced graphene oxide (r-GO) support and the development of acceptor energy levels dramatically enhanced electron-hole pair separation and transportation [18,34,35,36,37].

In this study we report the fabrication of a new photocatalyst using ilmenite sand and Ceylon graphite as the raw materials. Ilmenite is a naturally available mineral used mainly to isolate pure TiO_2_. In our previous studies we reported the synthesis of a wide range of nanocomposites using the acid leachate of ilmenite sand. Those nanocomposites, composed of α-Fe_2_O_3_/TiO_2_-Rutile/TiO_2_-Anatase, TiO_2_/Fe_3_C/Fe/Fe_3_O_4_/Graphitic Carbon, α-Fe_2_O_3_/Fe_2_TiO_5_/TiO_2_ and Fe_2_TiO_5_/TiO_2,_ have functioned as efficient photocatalyst nanocomposites [32,38,39]. The Fe_2_TiO_5_ semiconductor has recently gained prominence because of its narrow bandgap (Eg = 2.2 eV), and higher energy level conduction band minimum (CBM = −0.77 eV) [40]. Despite the fact that Fe_2_TiO_5_ exhibits excellent photoexcitation efficiency under visible light irradiation, appropriate band gap re-engineering strategies need to be deployed to avoid charge recombination. 

During the past decade, 2D graphene-like r-GO has been used in many areas such as electronics, photonics, supercapacitors, and fluorescence quenching in biology-related fields, biomedicine, catalysts and batteries [41,42,43,44]. The three primary synthetic techniques for GO preparation are the Brodie, Staudenmaier, and Hummer processes and their modifications [45,46]. The improved Hummers technique is employed in this study because it incorporates oxygen as oxygenated functional groups into the graphene structure, for example epoxy, hydroxy and carboxy. As well, their polar properties make GO hydrophilic, dispersed and exfoliated in many solvents. Such produced GO coupled to Fe_2_TiO_5_/α-Fe_2_O_3_ and the resulting composite was reduced by ascorbic acid to fabricate Fe_2_TiO_5_/α-Fe_2_O_3_/r-GO/GO heterogeneous photocatalyst composite from natural ilmenite and graphite. The photocatalytic activity of the synthesized nanophotocatalyst was examined to assess the degradation of methylene blue under visible light.

## 2. Materials and Methods

### 2.1. Chemicals and Materials

Ilmenite sand with a particle size of 100–200 µm was supplied by Lanka Mineral Sand (Pvt) Ltd. Graphite was obtained from Bogala, Sri Lanka. Hydrochloric acid (37%, HCl) and 2-propanol (99.95%, C_3_H_7_OH) were purchased from Sigma-Aldrich, St. Louis, MO, USA. Ammonium hydroxide (29%, NH_4_OH) was purchased from Merk & Co., Inc., Rahway, NJ, USA. Ferrous sulfate heptahydrate (99.5%, FeSO_4_.7H_2_O) and L-ascorbic acid (C_6_H_8_O_6_) were sourced from Sisco Research Laboratories in India. Methylene Blue was bought from Daejung Chemical & Metal Co., Ltd. (Shiheung, South Korea). All reagents used were analytical grade and solutions were prepared using ultra-pure water.

### 2.2. Dissolution of Ilmenite Sand and Precipitation

Washed and dried ilmenite sand was leached in 200 mL of concentrated HCl acid for 6 h at 110 °C and subjected to vigorous stirring under refluxing conditions. In a solid–liquid separation funnel, the leached slurry was allowed to cool and settle into layers. Orange colored supernatant was collected separately and is called the acid leachate. Oxygen gas was bubbled at 1 bar pressure overnight to oxidize ferrous ions to ferric ions. The homogenized mixture was filtered through a Whatman filter paper to separate the filtrate and solid residue. ICP-MS analysis examined the Fe and Ti concentrations in the leachate. The resulting solution was transferred to a two-neck round bottom flask, and the reaction mixture was bubbled with nitrogen gas for 1 h. In the nitrogen environment, 0.0237 mol FeSO_4_.7H_2_O was added to the reaction mixture. NH_4_OH was added dropwise with a continuing purge of nitrogen gas until the pH of the reaction mixture reached 10. The black slurry was then aged for another 12 h before being completely cleansed with ultra-pure water until the washings were chloride-free and neutral. The dried black color powder was calcined at 800 °C [32]. Powder that is not calcined is referred to as “amorphous TF”, while powder that is calcined at 800 °C is known as TF.

### 2.3. Preparation of Graphene Oxide

The improved Hummer’s method served to synthesize graphene oxide (GO) from natural graphite powder [46,47]. Graphite powder (3.0 g) and 12.0 g KMnO_4_ were homogenized together through mechanical grinding. The acid combination was prepared by mixing conc. H_2_SO_4_ (360 mL) and conc. H_3_PO_4_ (40 mL) at a ratio of 9:1 and kept in an ice bath to lower the temperature to prevent the exothermic reaction that occurs when the acid mixture is treated with the graphite and KMnO_4_ mixture. This mixture was treated with the acid mixture and the subsequent dark yellowish green solution was stirred for 12 h at 55 °C. The temperature of the mixture was allowed to cool down to room temperature before adding 3 mL of 30% hydrogen peroxide (H_2_O_2_); after that the stirring was continued for 5 min. Ice cubes controlled the subsequent exothermic reaction, and the final solution had a yellowish orange color. The obtained solid was washed with deionized water until the pH reached neutral and negative for SO_4_^2−^ ions. Finally, the collected material was dried at 60 °C for 12 h and is abbreviated as GO in this paper. 

### 2.4. Synthesis of Photocatalysts

GO powder was ultrasonically dispersed in ultra-pure water maintaining the GO/TF weight ratio at 10%. Then, the respective amount of TF was added to the GO suspension and further ultrasonicated for 30 min. The produced suspension was subjected to hydrothermal treatment at 150 °C for 8 hr. Then, 50 mL of L-ascorbic acid (0.1 M) solution was added to the mixture and stirred at 60 °C for 1 h. Finally, the obtained precipitate was washed with water and 2-propanol followed by drying at 60 °C. This material is referred to as (0.1)r-GOTF in this paper. Similarly, (0.2)r-GOTF and (0.3)r-GOTF materials were fabricated by maintaining the percentages of GO with reference to TF as 20% and 30%, respectively.

### 2.5. Photocatalytic Measurements

The photodegradation of MB served as the model reaction to evaluate the photocatalytic performance of the synthesized nanocomposites. TF catalyst (50 mg) was dispersed in 50 mL of MB (20 mg/L) solution and shaken in the dark for 180 min until adsorption–desorption equilibrium was achieved. Aliquots were withdrawn at desired time intervals and absorbance readings were recorded. After that, the solution was irradiated using a 50 W visible light generated by a LED source (the photon flux at the reactor was 1240 Wcm^−2^). Absorbance readings of the aliquots withdrawn at specific time intervals were recorded.

### 2.6. Materials Characterization

The Advanced Bruker X-ray diffraction (XRD) system investigated the crystalline structure of the produced samples. The X-rays were produced using a CuK-α anode and a 30-mA current at 40 kV voltage. The diffractograms were created using a 2°/min scanning rate in the 5–80° range, and the samples were analyzed using X’Pert High Score Plus (PANalytical) software. A Bruker Senterra Raman microscope spectrophotometer did the Raman analysis. A High-resolution transmission electron microscope (HR-TEM) and Zeiss field emission scanning electron microscope examined the morphology of the produced nanocomposites. Oxford Instruments energy dispersive X-ray (EDX) was used to acquire EDX spectra. X-ray fluorescence (XRF) determined the chemical composition of the samples using a HORIBA Scientific XGT-5200 X-ray analytical microscope with an Rh anode X-ray tube operating at a maximum voltage of 50 kV. Thermo Scientific^TM^ ESCALAB Xi^+^ X-ray Photoelectron Spectrometer (XPS) was utilized to examine the surface nature of the fabricated materials. The diffuse reflectance spectra (DRS) of the powder samples were analyzed using a Shimadzu 1800 UV/Visible spectrophotometer armed with a precision Czerny-Turner optical system. Measurements were made using a bandwidth of 1.0 nm and a wavelength range of 400–750 nm. Thermo Scientific X-Series 2 Inductively Coupled Plasma Mass Spectrometer (ICP-MS), Thermo Fisher Scientific, Waltham, MA USA helped to evaluate Fe^3+^ and Ti^4+^ concentrations of the acid leachate. Shimadzu UV-1990 double beam UV–Visible spectrophotometer was used to evaluate the absorbance of MB samples.

### 2.7. Determination of Antibacterial Activity against Escherichia coli

#### 2.7.1. Microbial Strain and Inoculum Preparation

The bacterial culture of *E. coli* used in the macro dilution assay was procured from Medical Research Institute, Sri Lanka. The initial inoculum was prepared by sub-culturing the pure cultures of *E. coli* in 250 mL of autoclaved nutrient broth media and then incubated at 37 °C for 24 h. 

To obtain accurate results it is crucial to standardize the bacterial cell number used for susceptibility testing. The density of the cell suspension was measured spectrophotometrically and with a 0.5 McFarland turbidity standard which is equal to 1 − 2 × 10^8^ CFU/mL as a visual yardstick. A 24 h aged bacterial culture was adjusted to obtain 5 × 10^5^ CFU/mL and the adjusted bacterial suspension was used immediately (within 30 min) to prevent subsequent changes in the cell count.

#### 2.7.2. Broth Dilution Assay 

The antibacterial activities of TF, (0.3)r-GOTF, (0.2)r-GOTF, (0.1)r-GOTF and r-TF composites were evaluated using the macro broth dilution method. The susceptibility assay of the synthesized material was carried out against *E. coli* in sterilized nutrient broth media. The viable count of *E. coli* bacteria was determined by the standard plate count method to clearly and easily discriminate the viability of bacterial cells after the treatment with fabricated nanocomposites. Bacterial culture and without nanocomposite functioned as the control.

A 24 h-aged *E. coli* culture adjusted with the spectrophotometer and 0.5 McFarland turbidity standard to obtain 5 × 10^5^ CFU/mL cell density was used for the broth dilution assay within 30 min to prevent any change in cell count. Suspensions of synthesized nanoparticles were prepared with deionized water by sonicating them at 50 °C for 40 min. Then, 1 mL of the prepared and adjusted bacterial culture was incorporated into 5 mL of sterilized nutrient broth media which contained 1 mL of 40 mg/mL nanocomposite suspensions. The positive control was prepared by inoculating 1 mL of adjusted bacterial suspension to 5 mL of sterilized nutrient broth media containing 1 mL of amoxicillin (40 mg/mL). Broth media inoculated with bacterial suspension yet free from nano-suspension was regarded as the growth control. The blank was prepared by adding the synthesized nanocomposites to sterilized nutrient broth media. The prepared solutions were placed in test tubes and incubated overnight for 24 h at 30 °C in a temperature-controlled shaker at 150 rpm. The inhibition of bacterial growth was determined through the spectrophotometric method. After 24 h, the optical density (O.D.) was measured at 600 nm using Shimadzu UV-VIS spectrophotometer.

Triplicates of each experiment were carried out. Each sample’s OD values and their mean values were calculated along with the standard deviation. The following formula was used to determine the percentage of growth inhibition.

% Inhibition = ((O.D.)_control − (O.D.)_test)/((O.D.)_control)(1)

where

(O.D.) control = Absorbance of the control sample
(O.D.) test = Absorbance of the test sample with the composites.

## 3. Results

The dissolution of ilmenite sand by hydrochloric acid produces iron and titanium chlorides as expressed in Equations (2)–(5). The ICP-MS quantified total Fe^3+^ and Ti^4+^ concentrations of oxygenated acid leachate, which were 5434 mg/L and 1078 mg/L, respectively. During bubbling of oxygen gas, all Fe^2+^ in acid leachate was oxidized and produced Fe^3+^. Hence, the total Fe concentration recorded by the ICP-MS analysis represents only Fe^3+^. The molar ratio of Fe^2+^ to Fe^3+^ was adjusted to 1:2 by adding 0.0237 mol of FeSO_4_.7H_2_O to the acid leachate under nitrogen environment. The amorphous TiO_2_/Fe_3_O_4_ composite after drying the precipitate obtained from the reaction of NH_4_OH and the acid leachate is shown in Equations (6)–(10). The XRD patterns of ilmenite sand and amorphous TiO_2_/Fe_3_O_4_ composite are shown in the supplementary information, specifically Figure S1a,b.

(2) $${\mathrm{FeTiO}}_{3}+4\;\mathrm{HCl}\;\to {\mathrm{Fe}}^{2+}+{\mathrm{TiOCl}}_{4}^{2-}+2{\;H}_{2}O$$

(3) $${\mathrm{FeTiO}}_{3}+4\;\mathrm{HCl}\;\to {\mathrm{FeCl}}_{2}+{\mathrm{TiOCl}}_{2}+2{\;H}_{2}O$$

(4) $${\mathrm{TiOCl}}_{4}^{2-}+\left(1+n\right){\;H}_{2}O\to {\mathrm{TiO}}_{2}.{n\;H}_{2}O+2{\;H}^{+}+4{\mathrm{Cl}}^{-}$$

(5) $${\mathrm{TiOCl}}_{2}+2{\;H}_{2}O\to H_{2}{\mathrm{TiO}}_{3}+2\;\mathrm{HCl}$$

(6) $${\mathrm{Fe}}^{2+}+2{\;\mathrm{OH}}^{-}\to \mathrm{Fe}{\left(\mathrm{OH}\right)}_{2}$$

(7) $${\mathrm{Fe}}^{3+}+3{\;\mathrm{OH}}^{-}\to \mathrm{Fe}{\left(\mathrm{OH}\right)}_{3}$$

(8) $$\mathrm{Fe}{\left(\mathrm{OH}\right)}_{3}\to \alpha -\mathrm{FeOOH}+H_{2}O$$

(9) $$\mathrm{Fe}{\left(\mathrm{OH}\right)}_{2}+2\;\alpha -\mathrm{FeOOH}\to {\mathrm{Fe}}_{3}O_{4}+2{\;H}_{2}O$$

(10) $$H_{2}{\mathrm{Ti}}_{3}O_{7}\to 3{\;\mathrm{TiO}}_{2}+H_{2}O$$

The α-Fe_2_O_3_/Fe_2_TiO_5_ heterojunction was formed after annealing of the TiO_2_/Fe_3_O_4_ composite at 800 °C since the solid-state reaction at the surface of the material occurred at 700–800 °C. Fe_3_O_4_ is converted to α-Fe_2_O_3_ when the annealing temperature reaches 600 °C [48] while α-Fe_2_O_3_ and TiO_2_ are expected to transform to α-Fe_2_O_3_/Fe_2_TiO_5_ once a further increase in the annealing temperature occurs [49]. The formation of Fe_2_TiO_5_ is shown in Equation (11) below:

(11) $$\alpha -{\mathrm{Fe}}_{2}O_{3}+{\mathrm{TiO}}_{2}\to {\mathrm{Fe}}_{2}{\mathrm{TiO}}_{5}$$

### 3.1. Morphological Analysis

#### 3.1.1. SEM and EDX Analysis

**Figure 1:**
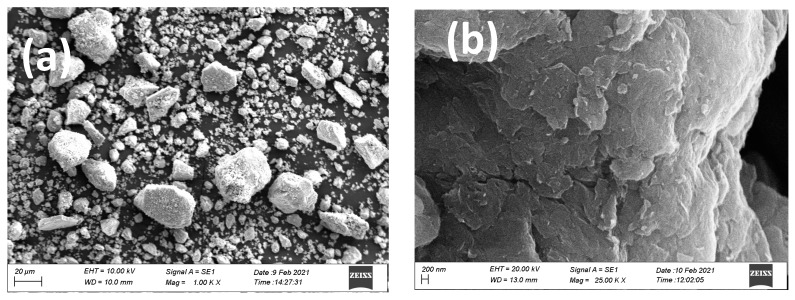

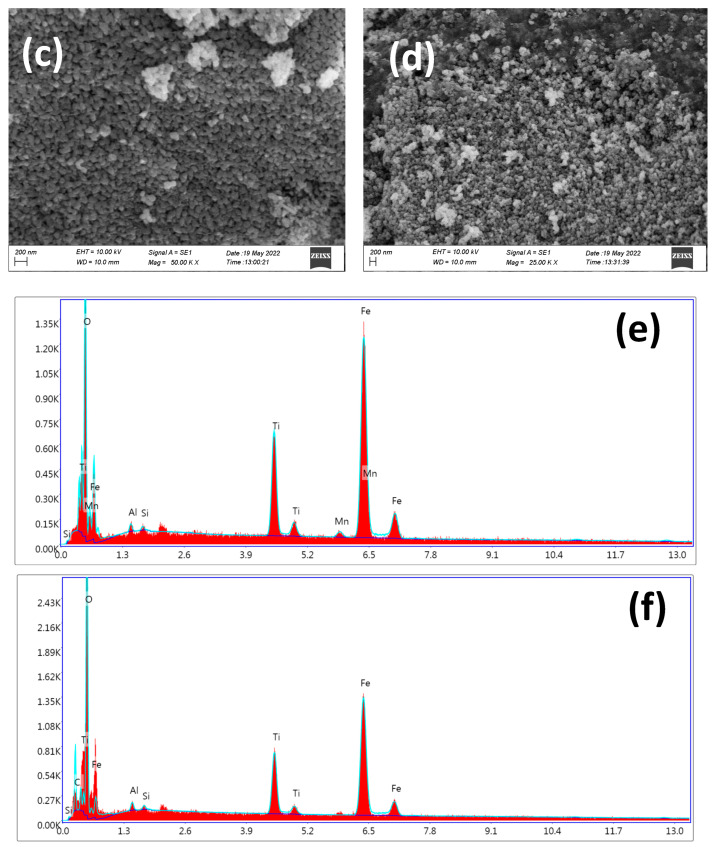
SEM images of (**a**) ilmenite sand (**b**) GO (**d**,**e**), (**c**) TF composite (**d**) (0.3)r-GOTF composite, EDX spectra of (**e**) TF composite and (**f**) (0.3)r-GOTF composite.

Surface morphological features of the fabricated nanocomposites were studied using scanning electron microscope and the collected images are shown in Figure 1. The SEM image of ilmenite sand (Figure 1a) reveals the macro nature of its particles with a heterogeneous particle size distribution. The crumpled and wrinkled lamellae structure arrangement is evident in the SEM image of GO developed from the oxidation of graphite (Figure 1b). The borders of individual sheets, including kinked and wrinkled portions, are distinct when these nanosheets are folded. This morphological feature of GO is very similar to the reported data [50]. Figure 1c shows the SEM image of TF nanoparticles. It is observed that the nanoparticles are fused at each end leading to aggregation, an outcome caused by Oswald ripening occurring during the calcination of the samples at elevated temperatures [51].

During Oswald ripening smaller nanoparticles dissolve and deposit as larger nanoparticles, thus achieving the most thermodynamically favorable configuration. The folded structure of GO disappears upon coupling with TF and reducing by L-ascorbic acid in the (0.3)r-GOTF nanocomposite, and the sample is dominated by nano-sized grains as shown in the SEM image (Figure 1d). Further, the surface morphology of TF has been altered after the reduction by L-ascorbic acid where almost monodispersed spherical nanoparticles were obtained compared to the TF shown in Figure 1c. EDX analysis was undertaken to identify the elemental composition of the samples. Figure 1e,f show the EDX spectra of TF and L-ascorbic acid treated (0.3)r-GOTF. EDX elemental composition results are tabulated in Supplementary Information (Table S1a,b), respectively). TF composite mainly consisted of Fe, Ti and O as the major constituents. Meanwhile the (0.3)r-GOTF nanocomposite is composed of C, Fe, Ti and O. The presence of C in (0.3)r-GOTF is mainly due to coupling with GO and r-GO. 

#### 3.1.2. TEM Analysis

**Figure 2:**
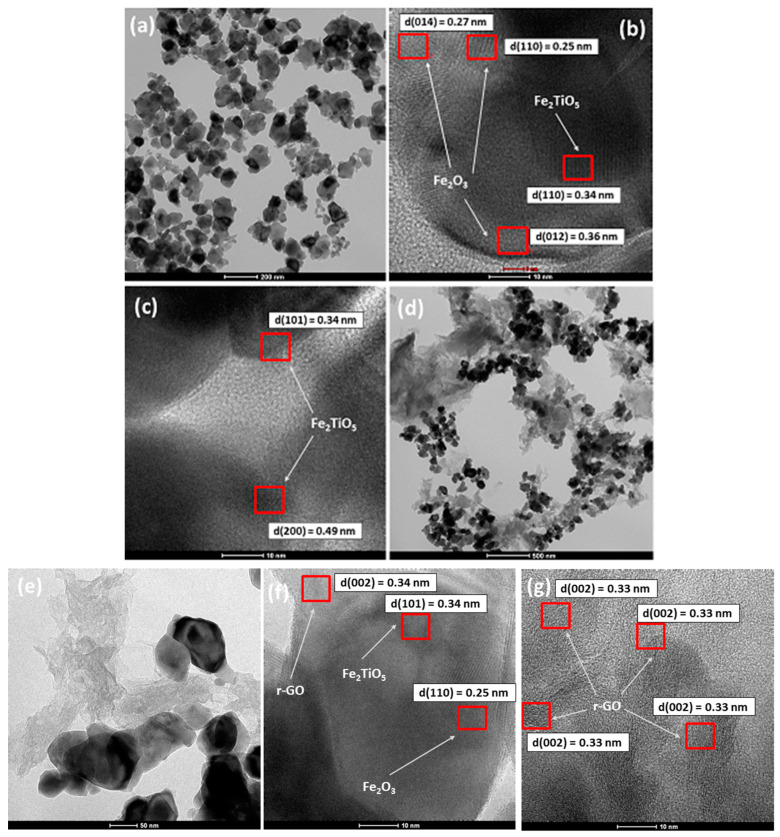
Bright-field TEM image of (**a**) TF, (**b**,**c**) HR-TEM images of (**d**,**e**) bright field TEM image of (0.3)r-GOTF, (**f**,**g**) HR-TEM images of (0.3)r-GOTF.

The inner structural and morphological features of TF and (0.3)r-GOTF composites were analyzed by TEM as shown in Figure 2. Bright field TEM image of TF (Figure 2a) shows spherical and irregular-shaped nanoparticles in the 60–100 nm range, and they are fused with each other forming aggregates being consistent with the SEM image of TF (Figure 1c). Fused nanoparticles are formed at high temperatures due to Oswald ripening. TiO_2_ and Fe_3_O_4_ nanoparticles formed at room temperature are converted to α-Fe_2_O_3_ and Fe_2_TiO_5_ at 800 °C and nanoparticle fusion forms a junction between α-Fe_2_O_3_ and Fe_2_TiO_5_, making possible the formation of α-Fe_2_O_3_/Fe_2_TiO_5_ heterojunctions. The HR-TEM images of TF composite are shown in Figure 2b,c, indicating atomic layer arrangement of varying phases. 

Interlayer distance values illustrated in Figure 2b 0.27, 0.25 and 0.36 nm correspond to the (014), (110) and (012) crystalline planes of α-Fe_2_O_3_, while (101) atomic plane of Fe_2_TiO_5_ is represented by 0.34 nm interlayer distance [52]. Further, (101) and (200) crystalline planes of Fe_2_TiO_5_ are exhibited in Figure 2c with corresponding atomic layer distances of 0.34 and 0.49 nm, respectively [49]. Crystalline planes can result in distinct electrochemical and catalytic characteristics [52]. High-index crystal planes of α-Fe_2_O_3_ nanocrystals are more susceptible to surface imperfections and have greater surface energy than low-index crystal planes [53]. As a result, the exposed crystal surfaces can drive photocatalytic activity. The selected area electron diffraction (SAED) patterns of TF are illustrated in Figure 2d. They appear to have scattered bright diffused rings, indicating the existence of a α-Fe_2_O_3_ structure, while the dark less intense patches suggest the presence of Fe_2_TiO_5_ in polycrystallinity [54,55].

The bright field TEM image of (0.3)r-GOTF nanocomposite shown in Figure 2e reveals that the bulk of Fe_2_O_3_/Fe_2_TiO_5_ nanoparticles are heterogeneous distributed on GO matrix. The higher magnification image of the same sample (Figure 2f) shows the aggregated large nanoparticles on GO support with some areas of GO having no nanoparticles, thus confirming the heterogeneous distribution. Further, it revealed that the folded nanosheet structures are randomly arranged as the r-GO and closely packed with the near boundaries of TF composites. HR-TEM images of the (0.3)r-GOTF are depicted in Figure 2g,h. The atomic planes with interlayer distances of 0.34, 0.25, and 0.33 nm in Figure 2g correspond to the (101), (110), and (002) crystalline planes of Fe_2_TiO_5_, α-Fe_2_O_3_, and r-GO, respectively, consistent with the d-spacing values calculated in the XRD analysis. Figure 2h shows the r-GO at the outer area of the composite. The lattice fringes with 0.33 nm of an interlayer distance which are uniformly distributed throughout the surface are assigned to the C(002) plane. Atomic planes with interplanar distance of 0.33 nm close to the metal nanoparticles suggest that the GO has been effectively reduced to r-GO even near the nanoparticles. Figure 2i depicts the selected area electron diffraction (SAED) patterns of (0.3)r-GOTF. The appearance of a set of diffraction patterns with sharp and clear diffraction patterns is denoted by the material’s polycrystallinity [54]. They appear to have scattered bright diffused rings, indicating the existence of a α-Fe_2_O_3_ structure, while the dark less intense patches suggest the presence of r-GO and Fe_2_TiO_5_ in polycrystallinity [54,55,56]. Previous studies have reported that most graphene sheets exhibited a single set of hexagonal diffraction pattern with clear and sharp diffraction spots [57].

### 3.2. XPS Analysis

**Figure 3:**
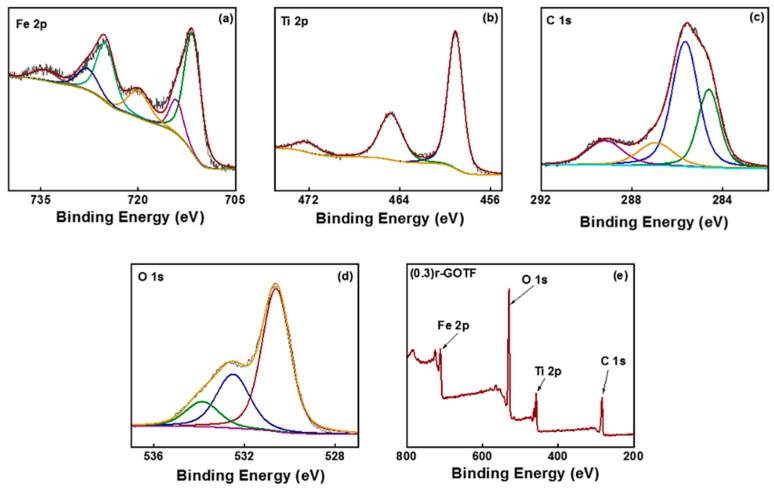
High-resolution XPS spectra of (**a**) Fe 2p of (0.3)r-GOTF, (**b**) Ti 2p of (0.3)r-GOTF, (**c**) C 1s of (0.3)r-GOTF, (**d**) O 1s of (0.3)r-GOTF, Survey spectra of (**e**) (0.3)r-GOTF.

X-ray photoelectron spectroscopy (XPS) emerged as a more surface-sensitive approach to describe the surface chemical states of fabricated nanocomposites. The higher resolution spectra of the elements and survey spectra of the synthesized composites are shown in Figure 3. All binding energies were corrected for the sample charging effect considering the binding energy of sp^2^ hybridized C=C as 284.6 eV. The higher resolution spectrum of Fe 2p of (0.3)r-GOTF is shown in Figure 3a while that of other composites are given in Supplementary Figure S2. The higher resolution spectrum of Fe 2p was mainly deconvoluted into two peaks as 2p_3/2_ and 2p_1/2_ and this generally indicated the spin orbital coupling. The peaks correspond to Fe 2p_3/2_ of the nanocomposite materials that appeared in the 711.84–712.06 eV range and the peak corresponding to Fe 2p_1/2_ appeared in the range of 725.28–726.03 eV, respectively [57]. The calculated binding energy differences are in the 13.64–13.73 eV range and satisfy the spin-orbit splitting of Fe 2p because Fe(III) is usually high-spin and exists in the form of Fe^3+^ [58]. The chemical environments of Fe^3+^ in α-Fe_2_O_3_ and Fe_2_TiO_5_ are different, although the oxidation number is the same. It can be observed that the resultant binding energies differ from those of the pure compounds, demonstrating the effective fabrication of Type II heterojunctions in the binary composites and altering the electronic environment of an individual element [59].

In the detailed analysis of Fe 2p_1/2_ and Fe 2p_3/2_ binding energies of fabricated nanocomposites, (0.3)r-GOTF exhibited by highest value in the above-reported range, values of 726.03 and 712.34 eV, respectively. The higher binding energy suggests an increment in the electron concentration of α-Fe_2_O_3_ and Fe_2_TiO_5_, despite the fact that this may further revoke the reduction in electron and hole recombination in composites [60,61]. Figure 3b shows the higher resolution spectrum of Ti 2p XPS spectra of (0.3)r-GOTF nanocomposite material, while the other composites are shown in Supplementary Figure S2. As shown in Figure 3b, Ti 2p is deconvoluted to three peaks at 459.1, 464.8, 472.5 eV, corresponding to the 2p_3/2_, 2p_1/2_, and satellite peak of 2p_3/2_ [32,39]. The spin-orbit splitting of the Ti 2p XPS spectrum of TF is = 5.7 eV, and its two binding energies of 459.1 and 464.8eV may be attributed to Ti^4+^ characteristic peaks, demonstrating the existence of Fe_2_TiO_5_ in the composite material [32,39]. The Ti 2p high resolution spectra of TF, (0.2)r-GOTF, (0.1)r-GOTF and r-TF materials do not indicate a considerable shift for calculated binding energy values. 

The high-resolution XPS C 1s spectra of the (0.3)r-GOTF is shown in Figure 3c and that of TF, (0.2)r-GOTF, (0.1)r-GOTF and r-TF are shown in Supplementary Figure S2. C 1s spectra of (0.3)r-GOTF and it depicts three peaks that appeared at 284.6 eV, 285.7, 287 and 289.2 eV representing, respectively, C=C, C–C and C=O [62]. Similarly, C 1s spectra of r-TF nanocomposite Supplementary Figure S2l exhibit identical behavior to the TF nanocomposite and C=C, C–C and C=O peaks appearing at 284.79, 285.79 and 288.91 eV, respectively. However, r-GO loaded samples reveal four significant peaks due to the presence of the C-O peak in GO samples. Moreover, sp^3^ C bonding is an important attribute of GO in producing good hydrophilic and wear resistance properties. This is despite the fact that we primarily focus on the fabrication of nanocomposites with the presence of the sp^2^ C bonding and sp^3^ C bonding fractions at the edge of r-GO/GO ultrathin films. 

For deeper analysis, C 1s high-resolution spectrum was deconvoluted in each sample using four Gaussian curves that correspond to sp2 carbon (sp^2^ C) bonding (~284.8 eV), sp^3^ carbon (sp^3^ C) bonding (~285.6 eV), C-O bonding (~287.1 eV), and C=O bonding (~289.4 eV) [38]. Furthermore, the calculated chemical composition of carbon materials is summarized in Table 1. The surface chemical percentage of C 1s has been calculated using the deconvoluted Gaussian curves peaks area under the curve. According to the XPS data in Table 1, (0.1)r-GOTF material shows the highest fraction of C=C (sp^2^ C) which was 22.54%. Consequently, the XPS results corroborated the Raman analysis results.

Figure 3d (p)–(t) shows the high resolution O 1s XPS spectra of (0.3)r-GOTF while the as-fabricated TF, (0.2)r-GOTF, (0.1)r-GOTF and r-TF nanocomposites are shown in Supplementary Figure S2. The spectra of TF, (0.3)r-GOTF, (0.1)r-GOTF and r-TF have been fitted to two deconvoluted peaks with binding energies of ~530.8 and ~531.86 eV with standard deviations of 0.11 and 0.16 eV, respectively [63]. The peak corresponding at ~530.8 eV was ascribed to the O^2−^ of Fe^3+^ and Ti^4+^ for α-Fe_2_O_3_ and Fe_2_TiO_5_, respectively [32,38,39]. As well, the peak appearing at 531.86 eV is ascribed to the OH. The O 1s high-resolution XPS spectra of (0.2)r-GOTF are represented in Supplementary Figure S2n), and it has been fitted into three deconvoluted peaks at binding energy values at 530.8, 532.36 and 534.04 eV, respectively [32]. In addition to the O^2−^ and OH peaks of Fe^3+^ and Ti^4+^ for α-Fe_2_O_3_ and Fe_2_TiO_5_, the peak appearing at 534.04 eV corresponded to the oxygen bound to carbon. The survey spectra for TF, (0.3)r-GOTF, (0.2)r-GOTF, (0.1)r-GOTF and r-TF nanocomposites are shown in supplementary Figure S2. C 1s, O 1s, Ti 2p and Fe 2p are observed as the major constituents. 

### 3.3. Raman Analysis

**Figure 4:**
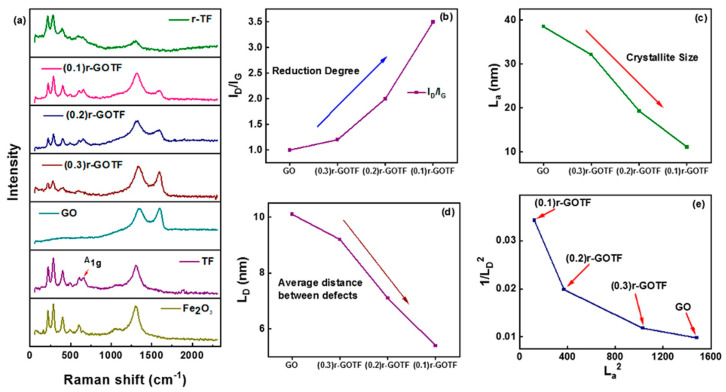
(**a**) Raman spectra of the synthesized composites, (**b**) I_D_/I_G_, (**c**) Variation of L_a_, (**d**) Variation of L_D_ and (**e**) 1/L_D_^2^ vs. L_a_^2^ of nanocomposites.

Nanocomposites were analyzed by Raman spectroscopy to study the crystal structure (Figure 4a–e). Hematite (α-Fe_2_O_3_) is a crystal form of iron oxide with a $D_{3d}^{6}$ crystal space group and seven characteristic Raman vibration modes [64]. Raman modes at 225 and 498 cm^−1^ are attributed to A1g modes and the bands at 247, 293, 299, 412, and 613 cm^−1^ are assigned to Eg modes of α-Fe_2_O_3_ [32,38,65] (Figure 4a). The Raman spectrum of TF composite shows typical Raman active modes of α-Fe_2_O_3_, with two A1g modes (225 and 498 cm^−1^) and Eg modes (247, 294, 299, 411, and 612 cm^−1^) [64]. However, some Eg modes of α-Fe_2_O_3_ in TF shifted by 1 cm^−1^ to the positive or negative side due to the fusion of α-Fe_2_O_3_ with Fe_2_TiO_5_. Further, characteristic Raman bands of Fe_2_TiO_5_ appeared at 199, 222, 334, 436, 658 cm^−1^, and 780 cm^−1^ in the Raman spectrum of TF_._ The Raman bands at 140 and 596 cm ^−1^ correspond to the B1g and A1g vibration modes of Fe_2_TiO_5_, respectively [38,54]. They appeared to have relatively low intensity, and this meant a small amount of Fe_2_TiO_5_ was present in the composite.

The crystallographic defects define the D-band, whereas the sp^2^ hybridized carbon atoms in the carbon network define the G-band [38]. The intensity ratio (I_D_/I_G_) is a quality measure of graphene derivatives based on the extent of flaws in their graphene-like structure [65]. In other words, the degree of functional and disordered sites in graphene-based materials is represented by the I_D_/I_G_ ratio. The variation of the I_D_/I_G_ ratios of GO, (0.3)r-GOTF, (0.2)r-GOTF and (0.1)r-GOTF is shown in Figure 4b. The I_D_/I_G_ ratio increases from 1.0 to 3.5 as reduction increases (GO ˂ (0.3)r-GOTF ˂ (0.2)r-GOTF ˂ (0.1)r-GOTF). Suggested here is that the creation of small-sized sp^2^ carbon domains or an increase in the fraction of edges is a result of reduction by the ascorbic acid.

Furthermore, as shown in Supplementary Figure S3, the D band of the nanocomposites has been blue-shifted away from the G band, further suggesting that reduction increased [63]. The rise in I_D_/I_G_ values with the increasing range of reduction in the nanocomposites strongly emphasizes that defects created in the reduced samples were due to the removal of oxygen groups. A reduction of full width at half maximum (FWHM) (Figure S3) in all the composites upon increasing the degree of reduction was observed as tabulated in Table 2; it was due to the reduction in the defective graphene-like domains. This finding confirms that the number of defect sites present in the (0.3)r-GOTF, (0.2)r-GOTF and (0.1)r-GOTF composites and the extent of reduction can vary by changing the amount of GO loaded. The presence of amorphous carbon and crystalline (graphene-like) materials is very important in photocatalytic applications. This is due to the simultaneous adsorption and photocatalytic performance. This phenomenon is further explained in Section 3.7.

When pure Graphite was oxidized to GO, the I_D_/I_G_ ratio of the resultant materials increased, indicating a higher level of structural disorder and more evident flaws in the graphene layers were the result of the oxidation process [66]. Following ascorbic acid reduction to produce a graphene-like composite, the I_D_/I_G_ relationship in the resultant material increased as reduction increased, showing that the exfoliation process integrated more flaws into the structure. The crystallite size (La) and distance between defects (L_D_) have been computed using Equations (12) and (13) (see below). In pure graphene, L_D_ is infinite but in extremely disordered graphene it is zero [67]. 

As demonstrated in Figure 4c, the L_a_ values of all GO-based composites improved as the reduction increased. This finding suggested that the average crystalline size of graphitic domains diminished when increasing the loading of GO. A similar pattern was seen with L_D_ (Figure 4d). Both the variations of L_a_ and L_D_ support the observation made with the variation of the I_D_/I_G_ ratio. Furthermore, Figure 4e depicts the variations in the density of the 0D defects (1/L_D_^2^) as a function of the crystallite area (La^2^) of (0.3)r-GOTF, (0.2)r-GOTF, and (0.1)r-GOTF composites. The number of 0D defects declines as the crystallite area expands, i.e., with decreasing GO loading, which is consistent with the behavior predicted by the variation of I_D_/I_G_.

(12) $$L_{a}\left(\mathrm{nm}\right)=\left(2.4\times 10^{-10}\right)\times \lambda_{l}^{4}\times {\left(\frac{I_{D}}{I_{G}}\right)}^{-1}$$

(13) $$L_{D}\left(\mathrm{nm}\right)=\sqrt{102\times {\left(\frac{I_{D}}{I_{G}}\right)}^{-1}\;}$$

where

$L_{a}=\mathrm{Crystallite}\;\mathrm{size}\;\left(\mathrm{nm}\right)$
$L_{D}=\mathrm{Average}\;\mathrm{distance}\;\mathrm{between}\;\mathrm{defects}\;\left(\mathrm{nm}\right)$
$\frac{I_{D}}{I_{G}}=\mathrm{Peak}\;\mathrm{intensity}\;\mathrm{ratio}\;\mathrm{between}\;D\;\mathrm{band}\;\mathrm{and}\;G\;\mathrm{band}\;$
$\lambda_{l}=\mathrm{Wavelength}\;\mathrm{of}\;\mathrm{laser}\;\left(\mathrm{nm}\right)$

### 3.4. XRD Analysis

**Figure 5:**
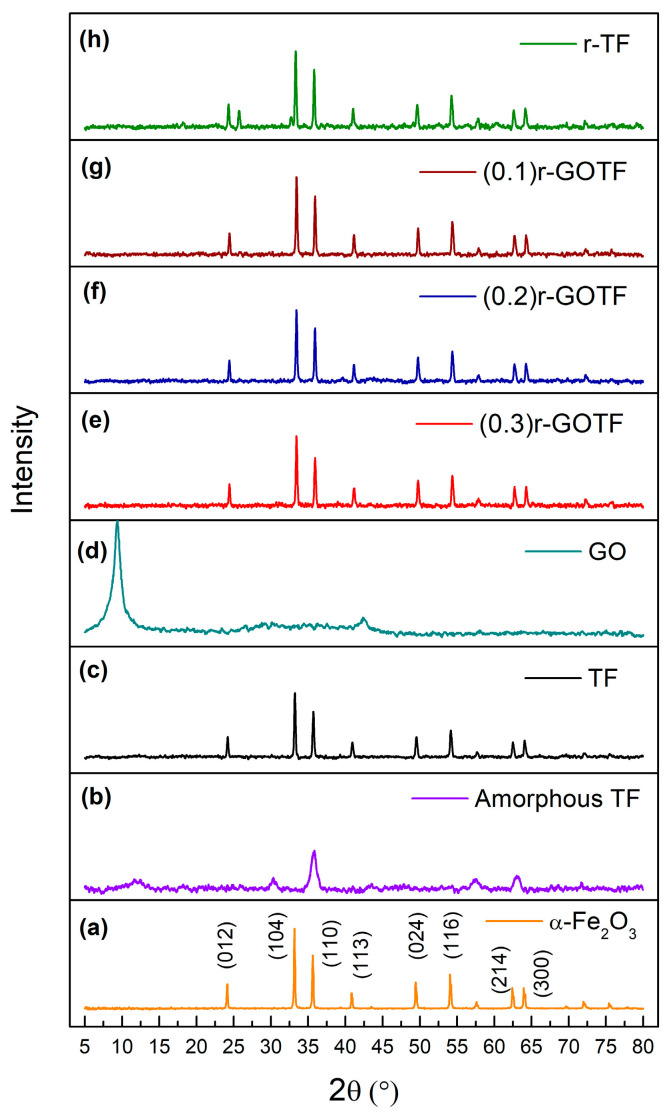
XRD patterns of (**a**) α-Fe_2_O_3_, (**b**) Amorphous TF, (**c**) TF, (**d**) GO, (**e**) (0.3)r-GOTF, (**f**) (0.2)r-GOTF, (**g**) (0.1)r-GOTF and (**h**) r-TF.

X-ray diffraction patterns were collected to further understand the crystal structure of the produced composites. The XRD pattern of the α-Fe_2_O_3_ annealed at 800 °C is shown in Figure 5a. The pattern consists of peaks at 24.42°, 33.40°, 35.86°, 41.12°, 49.68°, 54.32°, 57.86°, 62.64°, and 64.26° corresponding to the (012), (104), (110), (113), (024), (116), (018), (214), and (300) planes of α-Fe_2_O_3_ (ICDD DB card No. 01-079-1741) [32,38,39]. Figure 5b,c, show the XRD pattern of amorphous TF and the annealed TF nanocomposite, respectively. The crystallographic pattern of TF (Figure 5c) matches with ICDD DB card No. 01-079-1741 by suggesting the presence of α-Fe_2_O_3_. The diffraction pattern of TF composed by 2θ value centered on 24.18°, 33.22°, 35.69°, 40.94°, 49.54°, 54.16°, 57.68°, 62.51° and 64.09°, which corresponded to the (012), (104), (110), (113), (024), (116), (018), (214), and (300) of α-Fe_2_O_3_ [32,39]_._ However, the peaks corresponding to the Fe_2_TiO_5_ are not present in the XRD pattern. Fe^3+^ and Ti^4+^ concentrations found by ICP-MS analysis are 5434 mg/L and 1078 mg/L, respectively. FeO and TiO_2_ exist in weight percentages of 84.21% and 11.80%, respectively, in the TF material as revealed by the XRF analysis. Therefore, it is evident that the corresponding Ti present in the TF sample is comparatively low, producing a small amount of Fe_2_TiO_5_ leading to absence of peaks corresponding to Fe_2_TiO_5_ in the XRD pattern. The produced Fe_2_TiO_5_ could have been dispersed in TF which would have contributed to no peaks in the XRD pattern.

The XRD pattern of GO (Figure 2b) shows a sharp peak at 9.39° corresponding to the (001) atomic plane of graphene oxide [68]. The average crystalline size of the nanoparticles was calculated by the Debye-Scherrer Equation (14), and interlayer distance was calculated by Bragg’s Equation (15) [69]. The interlayer distance of GO was calculated as 0.94 nm and the crystallite size was found to be 7.13 nm; it consists of approximately 7 graphene layers. The XRD patterns of (0.3)r-GOTF, (0.2)r-GOTF and (0.1)r-GOTF are shown in Figure 5e–g, respectively, and appear to be identical to the XRD pattern of α-Fe_2_O_3_. The diffraction peaks corresponding to GO were not present because the folded structure of GO disappears during the ascorbic acid reduction, while the restacking of the nanosheets was observed when α-Fe_2_O_3_ nanoparticles are incorporated into r-GO sheets. The diffractions for the (012), (104), (110), (113), (024), (116), (214) and (300) crystalline planes of α-Fe_2_O_3_ were clearly observed with only a slight variation in diffraction angles [32,39].

The interlayer distance and crystalline size have been calculated based on the (104) plane of α-Fe_2_O_3_ and the results are summarized in Table 3. Figure 5h shows the XRD pattern of the r-TF nanocomposite, synthesized by reducing TF by ascorbic acid. It highlights the presence of Fe_2_TiO_5_ and α-Fe_2_O_3_, and the crystallographic data are consistent with ICDD DB card nos. 00-041-1432 and 01-079-1741, respectively. The XRD pattern of r-TF shows prominent diffraction peaks at 18.18°, 25.43° and 32.55° corresponding to the planes (200), (101) and (230), crystalline planes of orthorhombic Fe_2_TiO_5_ [32,39,70]. The diffraction peaks centered at the 2θ values of 24.32°, 33.44°, 35.76°, 41.19°, 49.71°, 54.39°, 57.92°, 62.71°, and 64.32° corresponding to the (012), (104), (110), (113), (024), (116), (018), (214), and (300) planes of α-Fe_2_O_3_ [64]. However, the XRD pattern of r-TF confirmed the surface-reducing effect of α-Fe_2_O_3_ TF by L-ascorbic acid. This is because the peaks corresponding to Fe_2_TiO_5_ appeared in the XRD pattern, suggesting that the material which covered Fe_2_TiO_5_ was removed upon treatment with L-ascorbic acid. The relative percentages of Fe_2_TiO_5_ and α-Fe_2_O_3_ were calculated based on the integrated peak area, while the r-TF was found to be composed of 84.75% of Fe_2_TiO_5_ (15.25%) and α-Fe_2_O_3_ (84.75%).

(14) $$L_{C}=\frac{K\lambda }{\beta \cos\theta }$$

(15) $$d=\frac{n\lambda }{2\sin\theta }$$

where,

$L_{C}-\mathrm{Average}\;\mathrm{crystallite}\;\mathrm{size}\;\left(\mathrm{nm}\right)$
β−Full width half maximum (rad)
θ−Diffraction angle (rad)
d−Interlayer distance (nm)
λ−Wavelength of X–ray source (1.5406 nm)
d−Interlayer distance (nm)

### 3.5. XRF Analysis

XRF analysis quantified the elemental composition of fabricated nanocomposites (Table 4). This investigation shows that ilmenite sand consisted of a number of minor impurities apart from the dominating Ti (48.87%) and Fe (42.81%) species including Al, V, Si, P, K, Ca, Cr, Mn, Zn and Zr. Similarly, in the other nanocomposites Ti and Fe are present as the major constituents and less than 4% of trace impurities such as Al, Si, Cr, Mn and Zn were detected. Impurities including V, P, K, Ca and Zr which were present in ilmenite were not detected. These impurities were removed during leaching in conc. HCl and during washing of the obtained precipitate. The amount of Fe in the fabricated nanocomposites is larger than that of the natural ilmenite, which is due to the addition of FeSO_4_.7H_2_O externally during the production. 

### 3.6. Optical Adsorption Properties (DRS Analysis)

**Figure 6:**
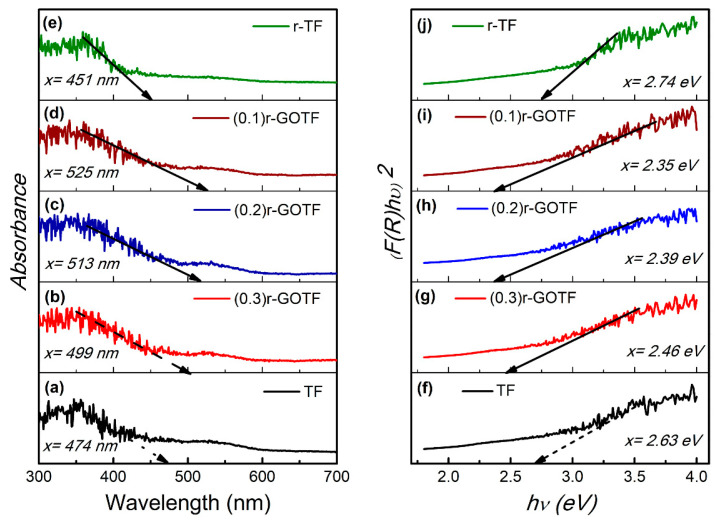
Adsorption vs. wavelength plot for (**a**) TF, (**b**) (0.3)r-GOTF, (**c**) (0.2)r-GOTF, (**d**) (0.1)r-GOTF and (**e**) r-TF, and direct band gap (n = 2) for (**f**) TF, (**g**) (0.3)r-GOTF, (**h**) (0.2)r-GOTF, (**i**) (0.1)r-GOTF and (**j**) r-TF.

Diffuse reflectance spectroscopy (DRS) is a technique used to study the interaction of light of "highly absorbing materials" such as metals, alloys and semiconductors [71]. The UV-Vis DRS optical absorption properties of the prepared nanocomposite materials are shown in Figure 6a–e. Tauc plots ${\left[F\left(R\right)h\nu \right]}^{n}$ vs. hν (photon energy) were plotted for direct transition using n = 2 as shown in Figure 6f–j to determine the band gaps of the fabricated nanocomposite materials. The Kubelka-Munk function, F(R), is given by Equation (17) and the band gap was determined using the Tauc plot.

(16) $$F\left(R\right)=\frac{\alpha }{S}=\frac{{\left(1-R\right)}^{2}}{2R}$$

(17) $${\left[F\left(R\right)h\nu \right]}^{n}=A\left(h\nu -E_{g}\right)$$

where,

α−Absorption coefficient(a.u)
S−Absorption coefficient(a.u)
$R-{\mathrm{Diffuse}\;\mathrm{reflectance}\;\mathrm{of}\;\mathrm{sample}\;\mathrm{divided}\;\mathrm{by}\;\mathrm{the}\;\mathrm{reflectance}\;\mathrm{of}\;\mathrm{the}\;\mathrm{BaSO}}_{4}$
$h-\mathrm{Planck}’s\;\mathrm{constant}\;\left(4.1357\times 10^{-15}\;\mathrm{eV}\;s\right)$
$E_{g}-\mathrm{Band}\;\mathrm{gap}\;\left(\mathrm{eV}\right)$
A−Proportional constant

The adsorption behaviors of the synthesized nanocomposites are shown in Figure 6a and the respective Tauc plots indicating direct transitions appear in Figure 6b. TF material exhibits considerable visible light absorption with an absorption edge at 474 nm and the corresponding band gap calculated was 2.63 eV. Similarly, (0.3)r-GOTF, (0.2)r-GOTF, (0.1)r-GOTF and r-TF nanocomposites exhibited visible light adsorption edges at 499, 513, 525 and 451 nm, respectively, corresponding to band gap values of 2.46, 2.39, 2.35 and 2.74 eV. TF nanocomposite is visible light sensitive due to the presence of Fe_2_TiO_5_ and Fe_2_O_3_. Similarly, all r-GO composed samples demonstrate considerable absorption increases in the visible light range compared to TF, thus indicating effective surface hybridization between these components. The determined direct band gap values were in the following order; (0.3)r-GOTF ˃ (0.2)r-GOTF ˃ (0.1)r-GOTF. As revealed by Raman analysis, (0.1)r-GOTF nanocomposite material possesses a larger amount of r-GO, compared to the (0.3)r-GOTF nanocomposite. (0.1)r-GOTF nanocomposite reaches a maximum absorption edge in visible light at 525 nm due to the enhanced level of reduction. According to the calculated bandgaps (0.1)r-GOTF is expected to show the highest photocatalytic activity, although (0.3)r-GOTF revealed the highest rate constant in photodegrading MB. This was due to the simultaneous adsorptive and photocatalytic properties, and this phenomenon is further explained in the section below.

### 3.7. Photocatalytic Degradation

**Figure 7:**
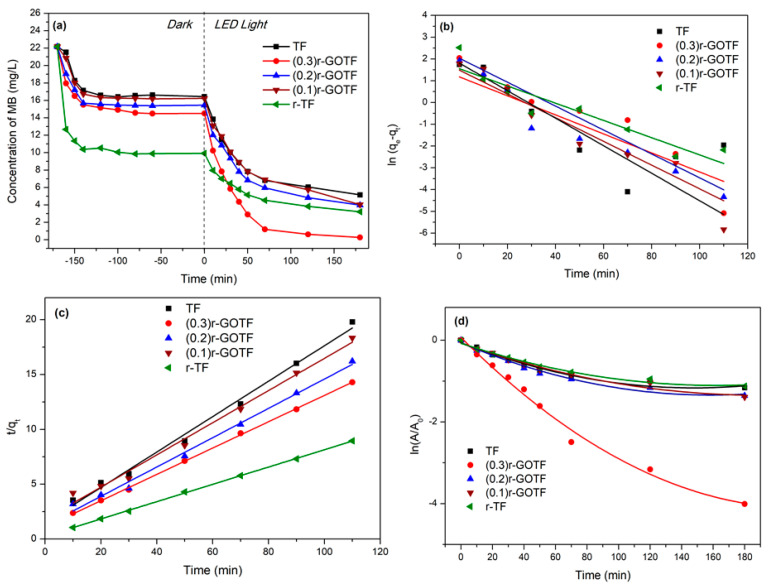

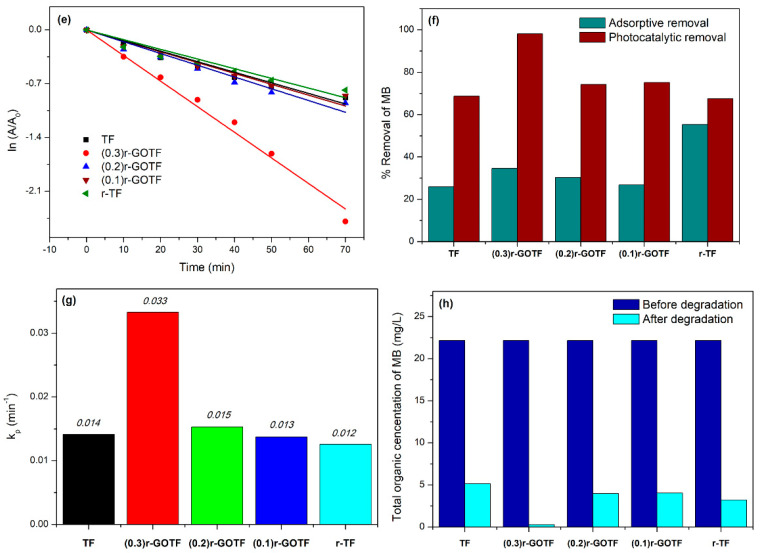
(**a**) Degradation of the MB concentration at different time intervals, (**b**) pseudo-first-order adsorption model of photocatalysts, (**c**) pseudo-second-order model of photocatalysts, (**d**) first-order photodegradation kinetics of MB degradation, (**e**) first-order photodegradation kinetics of MB degradation for first 70 min, (**f**) histogram of adsorptive and photocatalytic percentage removal of MB for photocatalysts, (**g**) histogram of first-order photodegradation rate constant for photocatalysts and (**h**) total organic concentration of MB in the photocatalysts system before and after degradation.

The visible light-driven photodegradation of fabricated nanocomposites was evaluated using MB. Experimental conditions were performed accordingly: catalyst loading (W = 50 mg), initial concentration of MB (C_0_ = 20 mg/L), volume of MB solution (V = 50 mL), temperature (T = 25 °C) and pH = 7. The adsorption of MB to the nanocomposites was evaluated for 180 min dark conditions and the photocatalytic activity was determined for 180 min under exposure to visible light. Adsorption-desorption equilibrium was reached within the first 100 min. Adsorption capacities obtained for TF, (0.3)r-GOTF, (0.2)r-GOTF, (0.1)r-GOTF, r-TF are 5.7, 7.7, 6.8, 6.0 and 12.4 mg/g, respectively as tabulated in Table 5. The overall adsorption and photocatalytic degradation process is shown in Figure 7a. The kinetics parameters of the pseudo-first-order and pseudo-second-order dark adsorption model are tabulated in Table 5. The best fitting model was chosen based on the linear regression correlation coefficient (r^2^) values. Equations (18) and (19) were used to express the linear form of the pseudo-first-order and pseudo-second-order adsorption kinetic models, respectively, and the respective plots are shown in Figure 7b,c [72,73]. It can be concluded that the adsorption of MB molecules to the composites follows the pseudo-second-order model as depicted by the higher correlation coefficient (r^2^) value (Table 5). The calculated $q_{e}$ values matched the experimental $q_{e,\;exp}$ values very well. This finding suggests that physical adsorption dominates the adsorption of MB dye molecules onto the composite surface:

(18) $$\ln\left(q_{e}-q_{t}\right)={\ln\;q}_{e}-k_{1}t$$

(19) $$\frac{t}{q_{t}}=\frac{1}{k_{2}q_{e}{}^{2}}+\left(\frac{1}{q_{e}}\right)t$$

where,

t=time (min)
$q_{e}=\mathrm{equilibrium}\;\mathrm{absorption}\;\mathrm{capacity}\;\left(\frac{\mathrm{mg}}{g}\right)$
$q_{t}=\mathrm{adsorption}\;\mathrm{capacity}\;\mathrm{at}\;\mathrm{time}\;t\;\left(\frac{\mathrm{mg}}{g}\right)$
$k_{1}=\mathrm{first}\;\mathrm{order}\;\mathrm{rate}\;\mathrm{constant}\;\left(L/\min\right)$
$k_{2}=\sec\mathrm{ond}\;\mathrm{order}\;\mathrm{rate}\;\mathrm{constant}\;\left(g/\mathrm{mg}*\min\right)$

Furthermore, the r-TF photocatalyst shows the highest second-order rate constant value of 0.024 g mg^−1^ min^−1^ while that of other composite materials, i.e., (0.1)r-GOTF, (0.2)r-GOTF, (0.3)r-GOTF and TF report smaller rate constant values, these being 0.012, 0.015, 0.013 and 0.018 g mg^−1^ min^−1^, respectively. The equilibrium adsorption capacity of photocatalysts follows the order of r-TF ˃ (0.3)r-GOTF ˃ (0.2)r-GOTF ˃ (0.1)r-GOTF ˃ TF. The enhanced adsorptive performance of r-TF could be attributed to the formation of a porous structure during the ascorbic acid treatment. This is despite the fact that increasing the loading of GO into photocatalysts meant that adsorptive performance improved. GO consists of oxygen functional groups, such as carboxyl groups and hydroxyl groups, bearing on the basal planes and edges of GO sheets. The (0.3)r-GOTF, (0.2)r-GOTF and (0.1)r-GOTF composites were subjected to the 1M ascorbic acid-based reducing process. 

During the reducing process, functional groups present in GO were eliminated to some extent by yielding graphene-like material on the surface near the metal oxide nanoparticles. According to the Raman analysis (Figure 4o–q and Table 2), (0.3)r-GOTF exhibits the highest L_a_ and L_D_ values, suggesting a large amount of defects compared to the other two samples of (0.2)r-GOTF and (0.1)r-GOTF. From Figure 4i and Table 2, it can be seen that compared with (0.1)r-GOTF and (0.2)r-GOTF, the I_D_/I_G_ ratio of (0.3)r-GOTF is further increased, indicating more defects are concentrated in the (0.3)r-GOTF sample. These results suggested the presence of more functional groups in (0.3)r-GOTF, compared to the (0.1)r-GOTF and (0.2)r-GOTF. Such a presence triggers strong electrostatic interaction between the negative surface and positively charged methyl nitride group [(CH_3_)_2_N^+^] of cationic MB dye molecule [72,74].

Photocatalytic degradation kinetics of organic molecules usually follow the Langmuir–Hinshelwood kinetics mechanism as shown in Equation (20) [75].

(20) $$-\frac{\mathrm{dC}}{\mathrm{dt}}=\frac{W}{V}I_{\propto }^{\beta }\frac{k_{r}K_{A}C_{t}}{1+K_{A}C_{0}}$$

The apparent kinetic content, denoted by $k_{\mathrm{app}}$, can be incorporated into the equation and simplified when using the following Equations (21) and (22).

(21) $$k_{\mathrm{app}}=\frac{W}{V}k_{r}K_{A}$$

(22) $$-\frac{\mathrm{dC}}{\mathrm{dt}}=r_{s}=I_{\propto }^{\beta }\frac{K_{\mathrm{app}}C_{t}}{1+K_{A}C_{0}}$$

Simplified differentiation Equation (23) was integrated by applying the boundary conditions to create the photocatalytic degradation rate equation, as shown in Equation (24).

(23) $$\ln\left(\frac{C_{t}}{C_{0}}\right)=-K_{\mathrm{app}}K_{A}t$$

(24) $$k=K_{\mathrm{app}}K_{A}$$

(25) $$\ln\left(\frac{C_{t}}{C_{0}}\right)=-\mathrm{kt}$$

where,

$K_{A}-\mathrm{Adsorption}\;\mathrm{equilibrium}\;\mathrm{constant}\;\left(a.u\right)$
$K_{r}-\mathrm{Kinetics}\;\mathrm{rate}\;\mathrm{constant}\;\left(a.u\right)$
$I-\mathrm{Light}\;\mathrm{intensity}\;\left({\mathrm{Wm}}^{-2}\right)$
∝and β−Normalization constant 
V−Volume of the reaction system (L)
W−Mass of the catalyst (mg)
$C_{t}-\mathrm{Concentration}\;\mathrm{at}\;\mathrm{time}\;t\;\left(\frac{\mathrm{mg}}{L}\right)$
$C_{0}-\mathrm{Initial}\;\mathrm{Concentration}\;\left(\frac{\mathrm{mg}}{L}\right)$
$k-\mathrm{Photocatalytic}\;\mathrm{kinetic}\;\mathrm{rate}\;\mathrm{constant}\;\left(\frac{1}{\min}\right)$
t−time (min)

Figure 7d,e show the kinetic parameters of MB degradation as a function of irradiation time exposed to a 1240 Wm^−2^ LED light (580 nm) source. Figure 7d indicates the overall degradation process as a second-order polynomial arrangement with two independent slopes for the complete degradation. Table 6 depicts the equations used to calculate polynomial regression and polynomial regression coefficient values. The polynomial regression coefficient values fitted the experimental data of the photodegradation process. The proposed mathematical arrangement clearly demonstrated that the degradation process was followed by a second-order polynomial fit with two intermediate rates determining elementary reactions. The first 70 min of the degradation process displayed a linear variation, establishing a starting rate slope for the total process. Despite this, the remaining 110 min slowed down the deterioration process by producing a second slope for the total degradation. This was due to the production of intermediate pollutants. Figure 7e depicts the photodegradation of MB over the first 70 min, and the estimated linear regression coefficients and rate constants are tabulated in Table 6. 

As shown in Figure 7e, the highest rate for the photodegradation of MB (0.033 min^−1^) was found with (0.3)r-GOTF because of the simultaneous adsorptive and photocatalytic mechanism, whereas r-TF degrades the slowest due to the creation of defective domains in the crystal lattices in α-Fe_2_O_3_. The impact of the defective domain formation effect during the ascorbic acid reduction of TF has been further explained in the Raman (Section 3.3) and XRD (Section 3.4) discussions. The calculated initial degradation rate constants of TF, (0.3)r-GOTF, (0.2)r-GOTF, (0.1)r-GOTF and r-TF were determined as 0.014, 0.033, 0.015, 0.013 and 0.012 min^−1^, respectively (Figure 7e). The rate constant of (0.3)r-GOTF is double that of (0.2)r-GOTF. Figure 7f exhibits the elimination of MB through the adsorption and photocatalysis employing various photocatalysts. MB was effectively removed via adsorption by all the catalysts in which r-TF dominated the activity (55.27%) and the catalysts’ performance varied as follows: r-TF ˃ (0.3)r-GOTF ˃ (0.2)r-GOTF ˃ (0.1)r-GOTF ˃ TF. 

The residual dye concentration after reaching the adsorption–desorption equilibrium was subjected to photocatalysis and the photocatalytic elimination of MB was varied as TF, (0.3)r-GOTF, (0.2)r-GOTF, (0.1)r-GOTF, and r-TF. Here, the respective percentage removals were 66.68, 98.18, 74.24, 75.13, and 67.55%, in which the (0.3)r-GOTF performed the best. Furthermore, as revealed by the TEM analysis of (0.3)r-GOTF the crystalline r-GO and non-crystalline GO domains were located at the edges of nanocomposites and located away from the metal nanoparticles. Moreover, the variation of I_D_/I_G_ ratio and L_D_ evident in Figure 4b,d obtained from the Raman interpretation showed that the (0.3)r-GOTF nanocomposite has more functional groups than (0.1)r-GOTF after the ascorbic acid reduction. 

The most important characteristic of adsorptive and photocatalytic performance is the extent of reduction. If the material has been greatly reduced, the absorptive performance may suffer due to decreased electrostatic attraction between the negatively charged photocatalyst surface and the positively charged MB molecule [76]. It is critical to precisely determine the appropriate degree of reduction by considering the total performance of the photocatalyst due to simultaneous adsorption and photodegradation. In summary, adequate defect concentration can significantly boost the r-GO/GO catalytic activity, whereas less defects will result in maximized charge carrier mobility. It may, however, limit the affinity towards the cationic MB dye molecule [77]. As a result, the recombination of excited electron-hole pair recombination centers may occur, limiting the samples’ photocatalytic activity. This concept further explains the photocatalytic activity of TF being greater than that of (0.1)r-GOTF. The results reported above further confirmed the (0.3)r-GOTF system’s better photocatalytic degradation, made possible by the appropriate degree of reduction. This has been further explained in Section 3.3.

**Figure 8:**
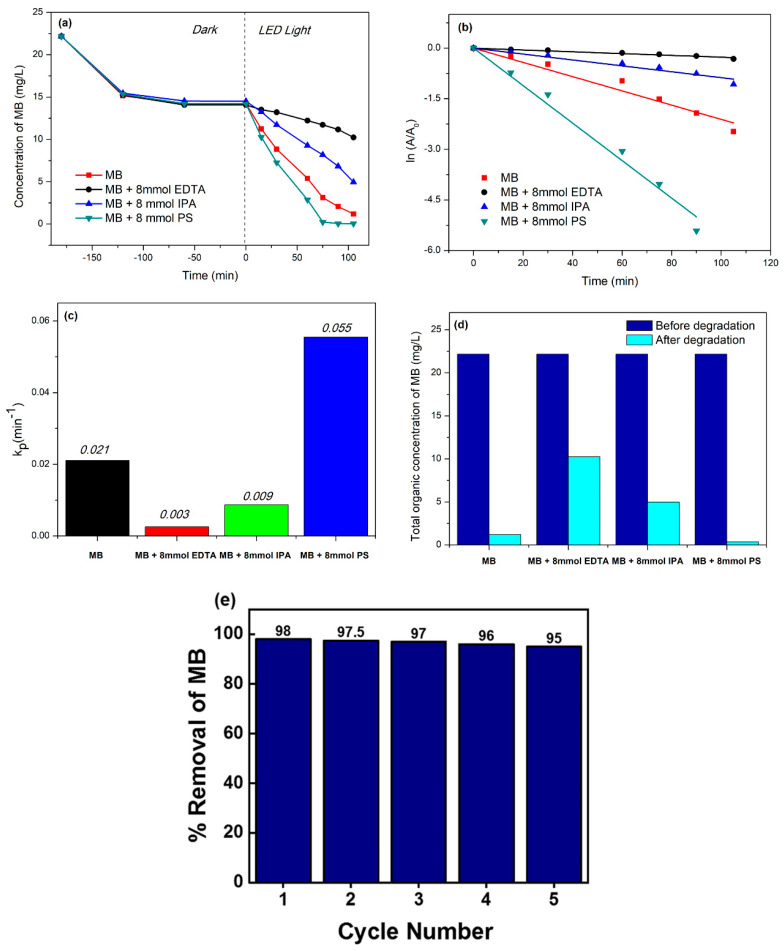
(**a**) Comparative degradation of the MB concentration in the presence of external reagents by (0.3)r-GOTF catalyst at different time intervals, (**b**) first-order kinetics degradation of MB by (0.3)r-GOTF catalyst, (**c**) histogram of first-order photodegradation rate constants for (0.3)r-GOTF catalyst in the presence of external reagents, (**d**) total organic concentration of MB in the photocatalysts system before and after the degradation and (**e**) reusability study of (0.3)r-GOTF.

Radical scavenging experiments were conducted to investigate the principal active radical species responsible for the degradation of MB in the presence of (0.3)r-GOTF. EDTA and IPA were added to the reaction mixture to test the impact of h^+^ and ^•^OH for the photodegradation of MB. Figure 8a depicts the variation of MB concentration with time showing the adsorption–desorption equilibrium followed by the photodegradation of MB upon exposure to visible light irradiation. Dark adsorption lasted for 180 min, and readings were collected for 100 min under visible light irradiation. Figure 8b depicts first-order kinetics whereas Figure 8c shows the estimated rate constants. No external reagents were added for the control experiment. Linear first-order rate constants in the control system and those with IPA and EDTA are 0.021, 0.009, and 0.003 min^−1^, respectively. As shown in Figure 8d, the concentrations of MB following the degradation process in the presence of EDTA, and IPA were 10.26, and 4.97, respectively, and the degradation efficiencies were 53.81, and 77.58%, respectively. Conversely, it was 94.63% in the control experiment.

The IPA and EDTA system substantially inhibited the ultimate degradation efficiency of MB, suggesting that both ^•^OH radicals and h^+^ are responsible for the degradation of MB where the contribution of h^+^ to the photodegradation of MB is greater than that of ^•^OH radicals. Effect of persulfate (PS) ions on the photodegradation of MB was studied. The PS-added system demonstrated a rapid drop in MB concentration, reaching 0.35 mg/L in 75 min. The PS system was 2.61 times quicker than the control system, degrading 98.42% of MB. PS increased visible light-driven photocatalysis by producing ^•^SO_4_^−^ radicals, as shown in Equations (26) and (27). Further, such produced ^•^SO_4_^−^ radicals produce ^•^OH with the reaction of OH^−^ as shown in the reactions (28) and (29). Both ^•^SO_4_^−^ and ^•^OH degrade MB increasing the rate of the reaction. Figure 8e shows the MB’s change of color after the degradation process.

(26) $$S_{2}O_{8}^{2-}+h^{+}\to S_{2}O_{8}^{•-}$$

(27) $$S_{2}O_{8}^{2-}+e^{-}\to {\mathrm{SO}}_{4}^{•-}+{\mathrm{SO}}_{4}^{2-}$$

(28) $${\mathrm{SO}}_{4}^{•-}+{\mathrm{OH}}^{-}\to {\mathrm{SO}}_{4}^{2-}+{\mathrm{OH}}^{•}$$

(29) $${\mathrm{SO}}_{4}^{•-}+S_{2}O_{8}^{2-}\to {\mathrm{SO}}_{4}^{2-}+S_{2}O_{8}^{•-}$$

Reusability of the best performing (0.3)r-GOTF catalyst (Figure 8e) was determined to figure out the suitability of the catalyst for industrial applications. It was evident that the drop of the conversion of MB was insignificant moving from cycle 1 to 5 and the slight drop is due to the loss of the material during washing and due to the coagulation of dye molecules in the porous system.

### 3.8. Mechanism of the Photocatalytic Activity

Based on the aforementioned results of different characterization analyses and light-driven degradation studies, Figure 6 proposes a probable synergistic mechanism of (0.3)r-GOTF. The proposed structure of (0.3)r-GOTF nanocomposite composed of Fe_2_TiO_5_/α-Fe_2_O_3_/r-GO/GO and the photocatalytic mechanism of nanocomposite is defined as follows. The band gaps for Fe_2_TiO_5_ and α-Fe_2_O_3_ found by diffuse reflectance spectroscopy are 2.25 and 2.20 eV, respectively. The conduction band and valance band potentials were determined using Equations (30) and (31), respectively.

(30) $$E_{\mathrm{CB}}=X-E^{c}-0.5{\;E}_{g}$$

(31) $$E_{\mathrm{VB}}=X-E^{c}+0.5{\;E}_{g}$$

where,

X—Absolute electronegativity of the semiconductor, which is defined as the geometric mean of the absolute electronegativity of the constituent atoms
E^C^—Energy of free electrons on the hydrogen scale
E_g_—The band gap of the semiconductor
E_CB_—Conduction band (CB) position
E_VB_—Valance band (VB) position

**Figure 9:**
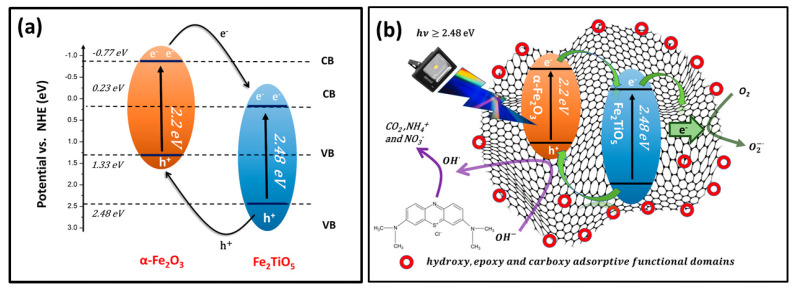
(**a**) Type II band alignment for Fe_2_TiO_5_ and α-Fe_2_O_3_ binary composite and (**b**) elucidated visible light-driven photodegradation mechanism of (0.3)r-GOTF.

The absolute electronegativity values of Fe_2_TiO_5_ and α-Fe_2_O_3_ are 4.78 and 4.76 eV, respectively. Furthermore, the energy of free electrons on the hydrogen scale is 4.5 eV vs. NHE. Table 7 lists the parameters used to calculate the VB and CB energy positions, and Figure 9a depicts the derived Type II band alignment in the Fe_2_TiO_5_ and α-Fe_2_O_3_ binary composite system.

When exposed to visible light, both α-Fe_2_O_3_ and Fe_2_TiO_5_ simultaneously generate electron-hole pairs because they are visible light sensitive semiconductors. In this scenario, r-GO can act as an electron reservoir, rapidly capturing and transporting photo-generated electrons from the CB of α-Fe_2_O_3_. Meanwhile, electrons at the CB of α-Fe_2_O_3_ migrate to the CB of Fe_2_TiO_5_ via the semiconductor heterojunction. This is due to the favorable potential difference in addition to the self-photogenerated electrons that would locate at the CB of Fe_2_TiO_5_. The electrons in the CB of Fe_2_TiO_5_ would also move into the conductive network of r-GO sheets, as a result of the coupling interfacial contact between r-GO and semiconductor heterojunction. Meanwhile the photo-generated holes in the VB of Fe_2_TiO_5_ move to the VB of α-Fe_2_O_3_. Therefore, Type II band alignment in (0.3)r-GOTF prevents photo-generated electron-hole pair recombination. 

The electrons in r-GO sheets react with $O_{2}$ to form $O_{2}^{•-}$ radicals. Similarly, ^•^OH are created at the VB of α-Fe_2_O_3_. (0.3)r-GOTF consists of a large amount of defective edges at the end of r-GO sheets. Those negative surface-charged oxidized species promote the rapid adsorption of MB dye molecules. The proximity of produced ^•^OH radicals to MB molecules on the catalyst surface facilitates the simultaneous breakdown of physisorbed MB as well as solution-containing MB. Consequently, including the Fe_2_TiO_5_/α-Fe_2_O_3_/r-GO/GO system confirms the improved degradation efficiency and suggested photocatalyst mechanism shown in Figure 9b. The following equations show the key intermediate reactions behind this overall degradation process of MB.

1. Absorption of efficient photons

(32) $$\propto -{\mathrm{Fe}}_{2}O_{3}+h\nu \to e_{\mathrm{CB}}^{-}+h_{\mathrm{VB}}^{+}$$

(33) $${\mathrm{Fe}}_{2}{\mathrm{TiO}}_{5}+\;h\nu \to e_{\mathrm{CB}}^{-}+h_{\mathrm{VB}}^{+}$$

2. Oxygen ionosorption:

(34) $${\left(O_{2}\right)}_{\mathrm{ads}}+e_{\mathrm{CB}}^{-}\to O_{2}^{•-}$$

3. Neutralization of ${\mathrm{OH}}^{-}$ groups by photo holes to produce OH^•^ radicals:

(35) $${\left(H_{2}O↔H^{+}+{\mathrm{OH}}^{-}\right)}_{\mathrm{ads}}+h_{\mathrm{VB}}^{+}\to h^{+}+{\mathrm{OH}}^{•}$$

4. Neutralization of $O_{2}^{•-}$ by protons:

(36) $$O_{2}^{•-}+H^{+}\to {\mathrm{HO}}_{2}^{•}$$

5. Transient hydrogen peroxide formation and dismutation of oxygen:

(37) $$2{\mathrm{HO}}_{2}^{•}\to H_{2}O_{2}+O_{2}$$

6. Decomposition of $H_{2}O_{2}$ and the second reduction of oxygen:

(38) $$H_{2}O_{2}+e_{\mathrm{CB}}^{-}\to {\;\mathrm{OH}}^{•}+{\mathrm{OH}}^{-}$$

7. Oxidation of the MB by ${\mathrm{OH}}^{•}$ radicals:

(39) $$\mathrm{MB}+{\;\mathrm{OH}}^{•}\to {\mathrm{MB}}^{•}+H_{2}O$$

8. Direct oxidation by reaction with holes:

(40) $$\mathrm{MB}+h_{\mathrm{VB}}^{+}\to {\mathrm{MB}}^{+•}\to \mathrm{degradative}\;\mathrm{products}$$

### 3.9. Antibacterial Activity against Escherichia coli

**Figure 10:**
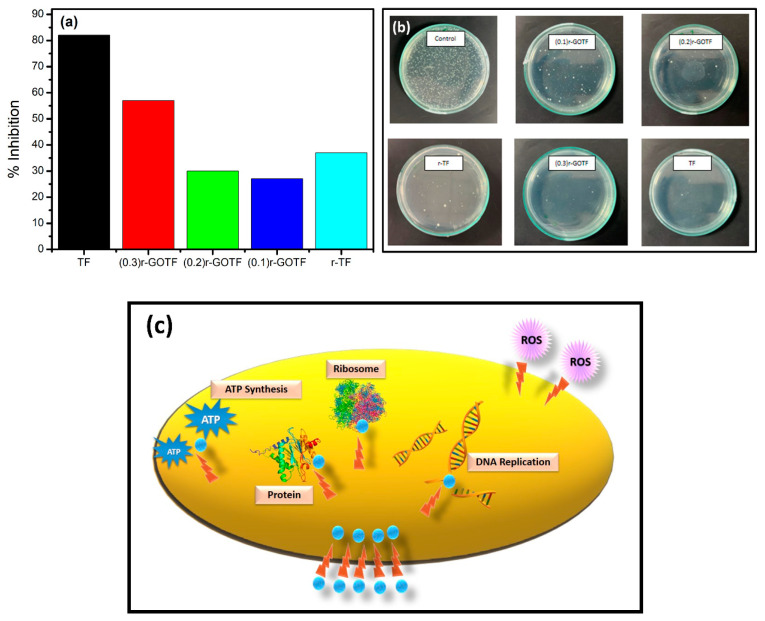
(**a**) Percentage inhibition of *E. coli* by fabricated nanocomposites, (**b**) sterilization performance of *E. coli* nanocomposites and (**c**) suggested photosterilization mechanism.

The antibacterial activity of TF, (0.3)r-GOTF, (0.2)r-GOTF, (0.1)r-GOTF and r-TF was evaluated for its effectiveness against Gram-negative bacteria *E. coli* (Figure 10). The maximum antibacterial activity against Escherichia coli resulted in the presence of TF leading to the removal of 82.5%. Antibacterial activity of GO-coupled nanocomposites followed the order of (0.3)r-GOTF ˃ (0.2)r-GOTF ˃ (0.1)r-GOTF while that of r-TF was lower than TF and (0.3)r-GOTF as shown in Figure 10a,b. The nanocomposites’ inhibition of bacteria growth is accompanied by four primary mechanisms (Figure 10c), namely: (1) the disruption of cell wall by nanocomposites; (2) migration of nanocomposites into the cells and interfering with the ribosomes, DNA replication, and interrupting ATP production; (3) perforation of the cellular membrane; and (4) disruption of cellular membrane by reactive oxygen species [38]. Upon excitation by light, the photon energy generates electron-hole pairs on the surface of nanocomposites, which produce ^•^OH radicals which can inhibit the growth of bacteria [78,79]. It can be deduced that the TF nanomaterial which exhibited less photocatalytic activity compared to (0.3)r-GOTF, has predominantly deterred the growth of bacteria via mechanisms (1), (2) and (3) with a minimum contribution from mechanism (4). This is due to the relatively small amount of reactive oxygen species generated as revealed in the photodegradation study. 

The antibacterial activity of the GO derivatives is affected by distinctive physicochemical features, i.e., orientation, size, shape, structural defects, and surface functional groups wielding a substantial impact on the biological response [80,81]. Moreover, the efficacy of the GO-coupled nanocomposites in discouraging bacterial cell growth is primarily influenced by their surface charge, size, and hydrophobicity [78]. The fabricated (0.3)r-GOTF nanocomposite was found to be between 30 nm to 80 nm, offering a high surface-to-volume ratio for the interaction with bacterial cells. A previous study proposed that relatively large GO sheets can cover bacterial cells more readily and once fully covered and biologically detached from their environment, cells cannot proliferate, resulting in cell viability loss observed in the subsequent colony counting test [78]. The r-GO formed following the reduction with ascorbic acid is hydrophobic in nature and the nanosheets tend to aggregate via π−π stacking.

Consequently, bacterial cell adsorption occurs rapidly and strongly on hydrophobic surfaces, while adhesion on hydrophilic substrates follows a reversible adhesion model [78,80]. The negative charge imparted by coupling TF with GO, followed by reduction from ascorbic acid, plays a key role in lowering the overall antibacterial activity of the GO-coupled nanocomposites. Weak electrostatic interaction between the negatively charged *E. coli* cell wall and negatively charged GO-coupled nanocomposite surface owing to the charge repulsion, accounts for the muted antibacterial activity when compared to that of TF. The GO-coupled nanocomposites mainly adhered to mechanism (4) as the amounts of radicals generated are comparatively higher than that of TF; this agrees with the overall adsorption and photocatalysis degradation activity against methylene blue. Among GO-coupled nanocomposites, (0.3)r-GOTF with the highest photocatalytic activity demonstrated the best antibacterial activity thus confirming the aforementioned statement.

However, the antibacterial activity of GO-coupled nanocomposites is lower than that of TF as the amount of nano-metal oxides present within the considered weight is low, signifying that mechanisms (1), (2) and (3) dominate mechanism (4) in preventing the growth of bacteria. The antibacterial activity of r-TF was poorer than that of TF, suggesting the antibacterial activity caused by α-Fe_2_O_3_ is comparatively superior to Fe_2_TiO_5_ since more of the Fe_2_TiO_5_ is exposed to bacterial cells than Fe_2_O_3_ upon reduction by ascorbic acid. Based on this, it could be concluded that inhibiting the growth of *E. coli* via mechanisms (1), (2) and (3) is more effective than mechanism (4). Furthermore, the size, surface charge and hydrophobicity influence the antibacterial activity of the fabricated nanocomposites.

## 4. Conclusions

Employing ilmenite and graphite powders as natural materials, we developed a facile ultrasonic and hydrothermal technique for fabricating the r-GO/GO/α-Fe_2_O_3_/Fe_2_TiO_5_ nanocomposite. Three distinct r-GO/GO/α-Fe_2_O_3_/Fe_2_TiO_5_ composites were created by varying the degree of reduction in GO domains, with ascorbic acid serving as the reduction agent. In this work, 30% loading of GO to α-Fe_2_O_3_/Fe_2_TiO_5_ photocatalyst ((0.3)r-GOTF) demonstrated better photodegradation efficiency of MB under visible light irradiation than pure α-Fe_2_O_3_/Fe_2_TiO_5_. The initial visible light-driven photodegradation rates of (0.3)r-GOTF and α-Fe_2_O_3_/Fe_2_TiO_5_ (TF) were 0.033 min^−1^ and 0.014 min^−1^, respectively. These enhancements can be attributed to the better charge transportation, separation and simultaneous adsorption and photodegradation, properties exhibited by (0.3)r-GOTF. Electrons photogenerated by α-Fe_2_O_3_ and Fe_2_TiO_5_ move over the conductive r-GO matrix, producing O_2_^•−^ and HO_2_^•^ radicals, while holes produce OH^•^ by interacting with the adsorbed water. Under visible light, these radicals successfully photodegraded MB molecules. The sacrificial agent investigation and DRS analysis both confirmed charge carrier recombination and light absorption augmentation. XRD, Raman, XPS and TEM analyses reveal the crystal structures and morphological features of fabricated nanocomposites. 

The TF and (0.3)r-GOTF composites were effective in inhibiting *E. coli* growth when exposed to visible light. The photosterilization and inhibition of TF (82.5%) was greater than (03)r-GOTF (56.5%). The r-GO produced after ascorbic acid reduction is hydrophobic in nature, and the nanosheets prefer to cluster via stacking. As a result, bacterial cell adsorption on hydrophobic surfaces happens quickly and robustly. The negative charge supplied by coupling TF with GO, followed by reduction from ascorbic acid, is critical in reducing the overall antibacterial activity of the GO-linked nanocomposites. Because of charge repulsion, the weak electrostatic connection between the negatively charged *E. coli* cell wall and the negatively charged GO-linked nanocomposite surface explains the diminished antibacterial activity when compared to that of TF. The present study describes a low-cost, simple approach for assembling r-GO into nanostructured semiconductors for use in removing MB dye from wastewater and photosterilization of *E. coli* for environmental remediation strategies.

## Figures and Tables

**Table 1:** Surface atomic composition of synthesized nanocomposites.

Material	Chemical Composition of Carbon (%)				
	C=C (sp^2^)	C-C (sp^3^)	C-O	C=O	C/O Ratio
TF	55.18	32.21	-	12.59	0.39
(0.3)r-GOTF	13.24	49.82	19.77	17.15	0.21
(0.2)r-GOTF	12.01	49.39	25.72	12.86	0.45
(0.1)r-GOTF	22.54	43.07	22.56	11.82	0.23
r-TG	45.73	41.09	-	13.17	0.30

**Table 2:** Raman characteristic peak positions, I_D_/I_G_ values of GO-based composite materials.

Material	Peak Position (cm^−1^)		FWHM (cm^−1^)		Peak Intensity Ratio			L_a_ (nm)	L_D_ (nm)
	D Band	G Band	D Band	G Band	D Band	G Band	I_D_/I_G_		
GO	1350.8	1589.6	149.1	84.2	806.6	777.5	1.0	38.5	10.1
(0.3)r-GOTF	1339.5	1588.5	135.1	83.6	229.7	186.4	1.2	32.1	9.2
(0.2)r-GOTF	1318.7	1572.7	134.4	78.1	277.1	138.6	2.0	19.3	7.1
(0.1)r-GOTF	1316.5	1585.5	133.9	73.1	352.7	100.4	3.5	11.1	5.4

**Table 3:** Structural properties of the composite analyzed by XRD crystalline planes.

Nanocomposite	Component	Peak Position (2θ)	Crystalline Plane (hkl)	Full Width at Half Maximum (FWHM) (β)	Integrated Peak Area	Crystalline Size (nm) (L_c_)	Interplanar Distance (nm) (d)
TF	Fe_2_O_3_	33.22	(104)	0.191	23.431	45.33	0.2694
GO	C	9.34	(001)	1.168	52.683	7.13	0.9461
(0.3)r-GOTF	Fe_2_O_3_	33.44	(104)	0.184	26.132	47.09	0.2677
(0.2)r-GOTF	Fe_2_O_3_	33.44	(104)	0.188	23.757	46.08	0.2677
(0.1)r-GOTF	Fe_2_O_3_	33.45	(104)	0.182	25.601	47.60	0.2676
r-TF	Fe_2_O_3_	33.33	(104)	0.186	24.712	46.57	0.2686
	Fe_2_TiO_5_	25.43	(101)	0.148	4.4415	57.47	0.349

**Table 4:** XRF analysis of ilmenite sand and fabricated nanocomposites.

Material	Al_2_O_3_ (%)	V_2_O_5_ (%)	SiO_2_ (%)	P_2_O_5_ (%)	K_2_O (%)	CaO (%)	TiO_2_ (%)	Cr_2_O_3_ (%)	MnO_2_ (%)	FeO (%)	ZnO (%)	ZrO_2_ (%)
Ilmenite	1.02	1.21	3.94	0.04	0.08	0.61	48.87	0.15	0.87	42.81	0.08	0.15
TF	1.04	-	1.21	-	-	-	11.80	0.17	1.48	84.21	0.04	-
(0.3)r-GOTF	1.07	-	1.35	-	-	-	11.37	0.17	1.77	82.23	0.04	-
(0.2)r-GOTF	0.73	-	1.07	-	-	-	11.46	0.16	1.24	85.31	0.03	
(0.1)r-GOTF	0.54	-	1.32	-	-	-	10.38	-	1.13	86.58	-	-
r-TF	0.91	-	0.98	-	-	-	11.66	0.16	1.24	85.72	0.05	-

**Table 5:** Kinetic parameters for dark adsorption of methylene blue onto nanocomposites.

Material	q_e, exp_ (mg g^−1^)	Pseudo-First-Order Model			Pseudo-Second-Order Model		
		q_e_ (mg g^−1^)	k_1_ (min^−1^)	$r_{1}^{2}$	q_e_ (mg g^−1^)	k_2_ (g mg^−1^ min^−1^)	$r_{2}^{2}$
TF	5.713	0.163	0.044	0.611	6.190	0.018	0.992
(0.3)r-GOTF	7.724	0.708	0.055	0.909	8.329	0.013	0.999
(0.2)r-GOTF	6.813	0.396	0.054	0.942	7.481	0.015	0.992
(0.1)r-GOTF	6.011	0.591	0.063	0.939	6.817	0.012	0.987
r-TF	12.421	0.438	0.039	0.857	12.674	0.024	0.999

**Table 6:** Linear and polynomial kinetic parameters for visible light photocatalysis.

Material	Polynomial Regression Analysis (180 min)			Linear regression Analysis (First 70 min)	
	Polynomial Equation	Polynomial Rate Constant k_p_ (min^−1^)	$r_{P}^{2}$	Initial Rate Constant k_p_ (min^−1^)	$r_{L}^{2}$
TF	$y=0.00005{\;x}^{2}-0.0149\;x$	0.008	0.996	0.014	0.994
(0.3)r-GOTF	$y=0.00008{\;x}^{2}-0.0667\;x$	0.025	0.987	0.033	0.998
(0.2)r-GOTF	$y=0.00005{\;x}^{2}-0.0161\;x$	0.009	0.978	0.015	0.997
(0.1)r-GOTF	$y=0.00003{\;x}^{2}-0.0134\;x$	0.009	0.989	0.013	0.991
r-TF	$y=0.00003{\;x}^{2}-0.0127\;x$	0.008	0.987	0.012	0.995

where, y = ($\ln(\frac{C_{t}}{C_{0}}$)), x = Exposure time (t)/(min).

**Table 7:** Parameters behind the calculation of VB and CB energy.

Semiconductor Oxide	Electronegativity (X)	E_g_ (eV)	Calculated CB Position (eV) (NHE)	Calculated VB Position (eV) (NHE)
Fe_2_TiO_5_	4.78	2.25	0.23	2.48
α-Fe_2_O_3_	4.78	2.20	−0.77	1.33

## Data Availability

The generated data during the study are available from the corresponding author upon request.

## Supplementary Materials

The following supporting information can be downloaded at: https://www.mdpi.com/article/10.3390/ma16010139/s1.
