# Supplementary material for: Fabrication of r-GO/GO/α-Fe2O3/Fe2TiO5 Nanocomposite Using Natural Ilmenite and Graphite for Efficient Photocatalysis in Visible Light
Source: Materials (Basel). 2022 Dec 23;16(1):139. doi: 10.3390/ma16010139 (PMC9821193; doi:10.3390/ma16010139)
Supplement: Supplementary file 1 [file materials-16-00139-s001.zip › materials-2091184-supplementary.pdf]

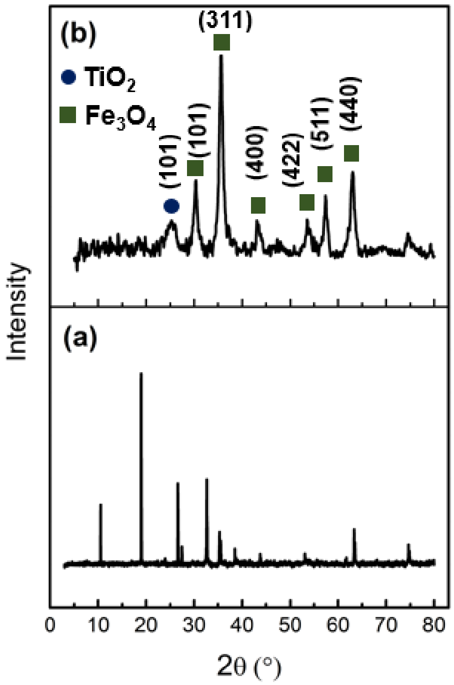

Figure S1. XRD pattern of (**a**) Ilmenite sand and (**b**) Amorphous TF.

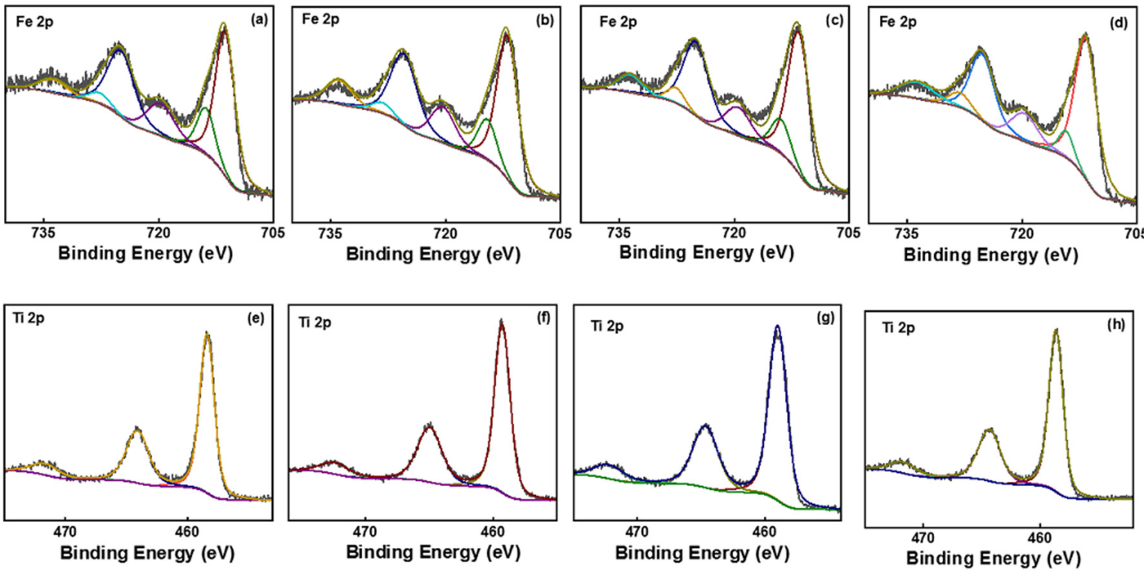

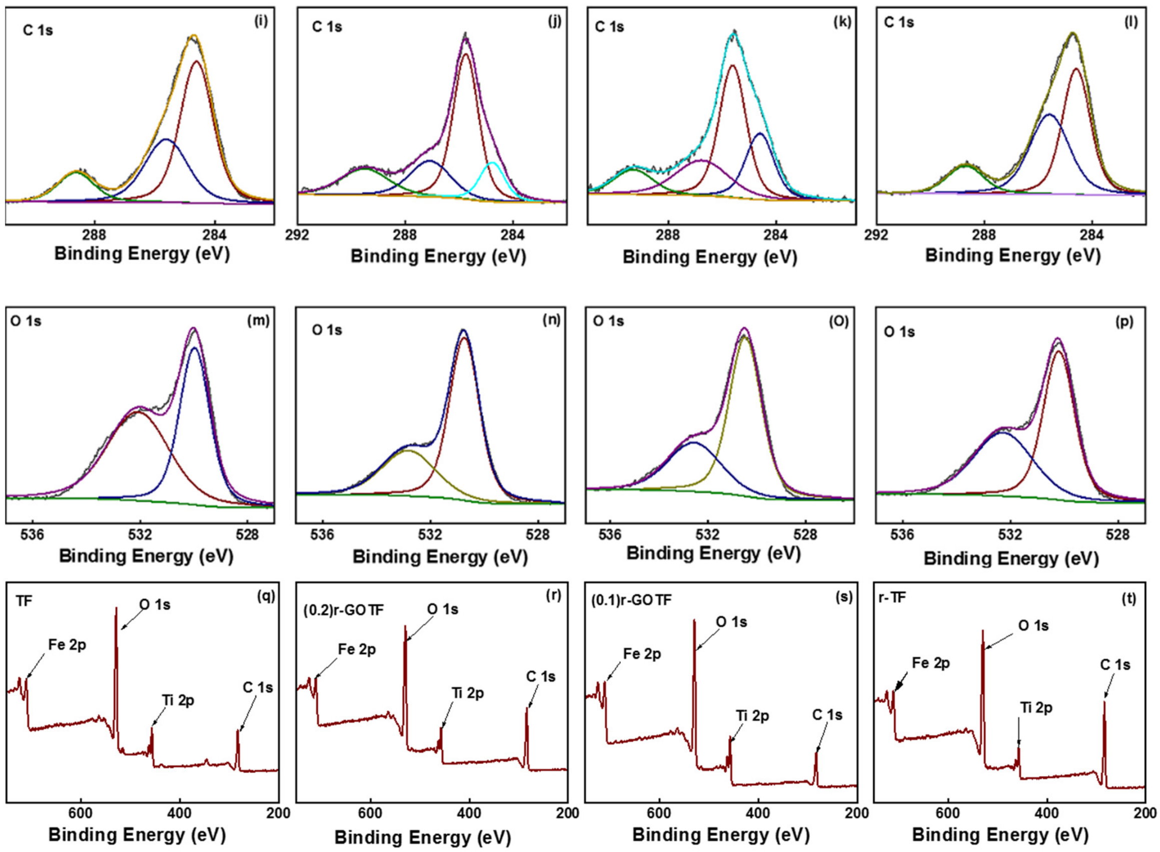

Figure S2. High-resolution XPS spectra of (**a**) Fe 2p of TF, (**b**) (0.2)r-GOTF, (**c**) (0.1)r-GOTF, (**d**) r-TF, Ti 2p of (**e**) TF, (**f**) (0.2)r-GOTF, (**g**) (0.1)r-GOTF, (**h**) Ti 2p of r-TF, (**i**) r-TF, (**j**) C 1s of TF, (**k**) C 1s of (0.2)r-GOTF, (**l**) C 1s of (0.1)r-GOTF, (**m**) C 1s of r-TF, (**n**) O 1s of TF, (**o**) O 1s of (0.2)r-GOTF, (**p**) O 1s of (0.1)r-GOTF Survey spectra of (**q**) TF, (**r**) (0.2)r-GOTF, (**s**) (0.1)r-GOTF and (**t**) r-TF.

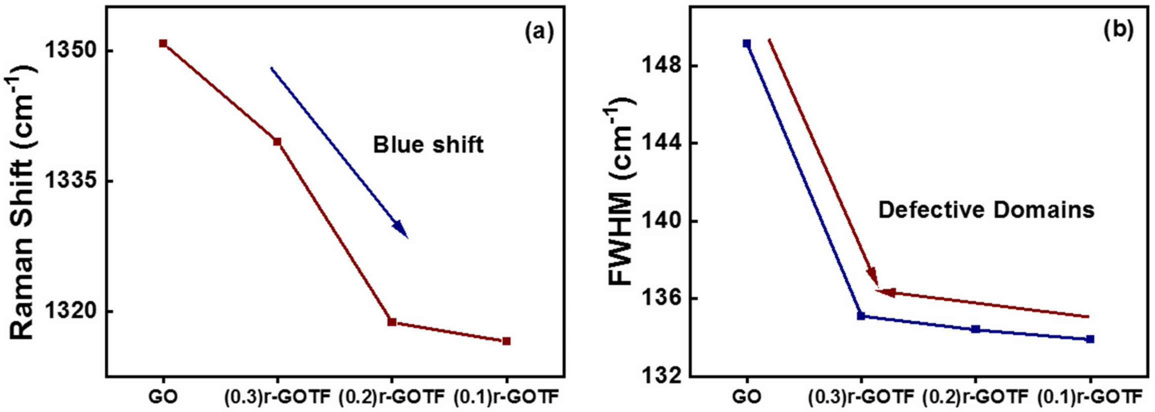

Figure S3. The variation of (**a**) D-band position, (**b**) FWHM of D-band in the Raman spectra of the composites.

Table S1. EDX elemental composition results of (a) TF and (b) (0.3)r-GOTF.

(a)								
**Element**	**Weight %**	**Atomic %**	**Net Int.**	**Error %**	**K ratio**	**Z**	**A**	**F**
O K	23.67	50.85	300.96	9.27	0.0850	1.1889	0.3020	1.0000
AlK	0.79	1.01	15.57	23.10	0.0035	1.0659	0.4123	1.0037
SiK	0.33	0.41	8.83	45.52	0.0020	1.0906	0.5399	1.0064
TiK	14.28	10.24	299.32	4.15	0.1486	0.9405	0.9852	1.1230
MnK	1.27	0.79	17.01	24.14	0.0121	0.9136	0.9806	1.0695

FeK	59.66	36.71	680.98	2.70	0.5496	0.9289	0.9882	1.0035

(b)

Element	Weight %	Atomic %	Net Int.	Error %	K ratio	Z	A	F
C K	9.19	19.50	92.01	10.68	0.0312	1.1741	0.2892	1.0000
O K	33.12	52.75	582.38	9.05	0.1097	1.1279	0.2935	1.0000
AlK	0.90	0.85	27.88	16.85	0.0042	1.0095	0.4574	1.0036
SiK	0.32	0.29	13.16	33.48	0.0020	1.0325	0.5864	1.0063
TiK	11.05	5.88	331.85	4.28	0.1093	0.8885	0.9952	1.1188
FeK	45.42	20.73	745.29	2.74	0.3993	0.8764	0.9965	1.0063
